# Supplementary material for: Preclinical and randomized phase I studies of plitidepsin in adults hospitalized with COVID-19
Source: Life Sci Alliance. 2022 Jan 10;5(4):e202101200. doi: 10.26508/lsa.202101200 (PMC8761492; doi:10.26508/lsa.202101200)
Supplement: Supplementary file 4 [file LSA-2021-01200_Supplemental_Data_1.pdf]

Multicenter, randomized, parallel and proof of concept study to evaluate the safety profile of three doses of Plitidepsin in patients with COVID-19 requiring hospitalization

COORDINATING INVESTIGATORS:

Dr Vicente Estrada
Hospital Clínico San Carlos, Department of Infectious Diseases

Dr Jesús Fortún Abete
Hospital Ramón y Cajal, Department of Infectious Diseases

Dr José Barberán
Hospital HM Montepríncipe, Department of Internal Medicine

PROTOCOL CODE: APLICOV-PC (APL-D-002-20)

EudraCT number: 2020-001993-31

SPONSOR: Pharma Mar, SA

Previous versions of the protocol/amendments (number and date):

- Version 1.0, dated 22/Apr/2020
- Version 2.0, dated 25/Apr/2020
- Version 3.0, dated 13/ May/2020
- Version 4.0, dated 18/Jun/2020
- Version 5.0, dated 13/ Aug/2020
- Version 6.0, dated 11/ Sep/2020

Current version (number and date):

- Version 7.0, dated 29/ Sep/2020

CONFIDENTIAL: The information and data included in this protocol contain trade secrets and classified or confidential information that is the property of the sponsor. No

person is authorised to make this information public without the written permission of the sponsor. These limitations will also apply to all information deemed classified or confidential that is provided in the future. This document may be disclosed and used by your team and collaborators, as necessary to carry out the clinical study.

1. PROTOCOL SUMMARY

1.1.Sponsor identification

Name:

Pharma Mar, SA

Contact Person:

Dr Maria José Pontes Valero
Associate International Medical Affairs Director
E-mail[redacted]
Tel:[redacted]
Fax:[redacted]

Dra. Belen Sopesen Veramendi,
Director of Corporate Affairs
e-mail:[redacted]
Tel:[redacted]

Postal address:

Avda. de los Reyes, 1.
Polígono Industrial “La Mina”.
28770 Colmenar Viejo.
Madrid, Spain.

Pharmacovigilance contact person:

Hervé Dhellot
EEA Qualified Person for Pharmacovigilance and Senior Pharmacovigilance Manager
e-mail[redacted]
Tel (24 hours):[redacted]
Tel:[redacted]
Fax:[redacted]

Postal address:

Avda. de los Reyes, 1.
Polígono Industrial “La Mina”.
28770 Colmenar Viejo.
Madrid, Spain.

Coordinating investigators:

Dr Vicente Estrada
Hospital Clínico San Carlos
Department of Infectious Diseases

Dr Jesús Fortún Abete
Hospital Ramon y Cajal
Department of Infectious Diseases

Dr José Barberán
Hospital HM Montepríncipe,
Department of Internal Medicine

1.2. Study title

Multicenter, randomized, parallel and proof of concept study to evaluate the safety profile of three doses of Plitidepsin in patients with COVID-19 requiring hospitalization

1.3. Protocol code

APLICOV-PC (APL-D-002-20)

1.4. Drug Research Ethics Committee (CEIm)

Drug Research Ethics Committee of HM Hospitales

1.5. Company responsible for monitoring

APICES
Av. Antonio López, 16 - 1ºA
28320 Pinto (MADRID)
Tel: [redacted]
Fax: [redacted]
E-mail: [redacted]

1.6. Investigational product

Plitidepsin

1.7. Clinical trial phase

Proof of concept

1.8. Study objectives

<u>Primary objective:</u>

To determine the safety and toxicological profile of plitidepsin at each dose level administered according to the proposed administration scheme in patients admitted for COVID-19.

Secondary objectives:

- To assess the efficacy of plitidepsin in patients with COVID-19 at the proposed dose levels by:
    - Change in SARS-CoV-2 viral load from baseline
    - Time until negative detection of SARS-CoV-2 by PCR
    - Cumulative incidence of disease severity. The evaluation will be based on:
        - Mortality
        - Need for invasive mechanical ventilation and/or ICU admission
        - Need for non-invasive mechanical ventilation
        - Need for oxygen therapy
- Selection of the recommended dose levels of plitidepsin for a phase II/III efficacy study

1.9. Study design

Multicentre, randomised clinical trial with three treatment arms.

Three cohorts of 9 patients will be included in the study. Each of the cohorts of 9 patients will include 3 arms (A, B, and C) at different dose levels with 3 patients in each arm, up to a maximum of 27 patients evaluable for safety.

The established dosage levels are as follows:

- Arm A) 1.5 mg of plitidepsin administered as a 1.5-hour infusion, once a day for 3 consecutive days.
- Arm B) 2.0 mg of plitidepsin administered as a 1.5-hour infusion, once a day for 3 consecutive days.
- Arm C) 2.5 mg of plitidepsin administered as a 1.5-hour infusion, once a day for 3 consecutive days.

**ARM**	**Dosage level**	**Daily dose (mg)**	**Total dose (mg)**
Arm A	1	1.5 mg x 3 days	4.5 mg
Arm B	2	2.0 mg x 3 days	6.0 mg
Arm C	3	2.5 mg x 3 days	7.5 mg

Recruitment will be sequential in each of the three arms. The first cohort of 3 patients in arms B and C (2.0 mg and 2.5 mg) will not open until the data have been analysed for the first 3 patients at the dose level immediately below (arms A or B) without observing any unacceptable toxicity, taking into account the data of the three patients included in each arm with a 12-day follow-up from the end of treatment (day +15 from the start of treatment).

When patients are to be included at more than one dose level (arm), patients will be randomised in a 1:1 or 1:1:1 ratio as appropriate.

The following criteria have been established to continue the inclusion of patients in the different arms:

- If 0-1 of the first three patients treated with the dose of 1.5 mg x 3 days experience any adverse event classed as grade 3 or higher within twelve days after administration of the last dose, three additional patients will be enrolled in the 1.5 mg arm and recruitment of the first three patients in the 2.0 mg arm will start. Recruitment in the 2.5 mg arm will be opened after it has been proven that only 0-1 of the three patients in the first cohort in the 2.0 mg arm have grade 3 or greater toxicity within twelve days of follow-up after administration of the last dose of 2.0 mg.
-
- If 2 or more patients in any of the arms present an adverse event classed as grade 3 or higher, the sponsor will interrupt the recruitment of new patients and will jointly assess with the Spanish Agency of Medicines and Medical Devices whether or not to continue recruitment in that arm and in arms with a higher dose.

Intra-patient dose change/increase is not allowed.

Once the 27 patients (9 at each dose level) contemplated in the study protocol have been included, an extension period will be opened to include 18 additional patients (6 at each plitidepsin dose level) to extend the collection of safety information on the different doses and additionally to collect efficacy data. After this extension period, data will be available for 15 patients at each plitidepsin dose level.

During the extension period, patients will be randomised in a 1:1:1 ratio as appropriate to each dose level (arm).

During the extension period of the study, if in any of the arms, 2 or more patients present an adverse event classed as grade 3 or higher related to plitidepsin, the sponsor will interrupt the recruitment of new patients at that dose level and higher, and will assess jointly with the Spanish Agency of Medicines and Medical Devises the convenience of continuing recruitment in that arm and in arms with a higher dose. Additionally, the sponsor will forward weekly follow

up records to the Spanish Agency of Medicines and Medical Devices, with the information of all the adverse event classed as grade 3 or higher, reported, whether or not related to plitidepsin, for review and joint assessment if necessary.

After the joint evaluation of the safety and efficacy data obtained in the present study between the sponsor and the Spanish Agency of Medicines and Medical Devices, the recommended dose level will be selected to evaluate the efficacy of the treatment in patients with COVID-19 in a subsequent randomised phase II/III study.

The final statistical analysis that will be the basis of the final report of the study will be done on the total population of 45 patients once the extension of the study has been completed.. The data of the first 27 patients will be analysed in an intermediate statistical analysis.

1.10. Disease under study

COVID-19 infection

1.11. Endpoints

<u>Primary endpoint:</u>

To determine the safety and toxicological profile of plitidepsin at each dose level administered in patients admitted for COVID-19, the following endpoints will be analysed:

- Frequency of the following ≥ grade 3 adverse events according to the NCI-CTCAE criteria v 5.0 at 3, 7, 15 and 31 days:
  - Haematological
    - Neutropenia
    - Thrombopenia
    - Anaemia
    - Lymphopenia
  - Non-haematological
    - CPK increase
    - ALT and/or AST increase
    - Total bilirubin or direct bilirubin increase
    - Neurotoxicity
    - QT-QTc interval prolongation
  - Other ≥ grade 3 adverse events
- Percentage of patients in which treatment cannot be completed and reasons.
- Percentage of patients with AEs and SAEs at days 3, 7, 15 and 31.

- Changes from baseline (day -1 or day 1 before administration of the study drug) in haematological and non-haematological parameters on days 3, 7, 15 and 31.
- Percentage of patients with ECG abnormalities on days 2, 3, 4, 5, 6, 7, 15 and 31.

Secondary endpoints:

- Change in SARS-CoV-2 viral load from baseline (day -1 or day 1 before administration of the study drug) measured at days 3, 4, 7, 15 and 31.
- Time until the detection of COVID-19 by PCR obtained from nasopharyngeal exudate or sample from the lower respiratory tract compared to baseline (day -2 or -1 or day 1 before administration of the study drug).
- Mortality at 7, 15 and 31 days.
- Percentage of patients requiring invasive mechanical ventilation and/or ICU admission at 7, 15 and 31 days.
- Percentage of patients requiring non-invasive mechanical ventilation at 7, 15 and 31 days.
- Percentage of patients requiring oxygen therapy at 7, 15 and 31 days.
- The recommended dose level will be selected after the joint evaluation of the safety and efficacy data obtained in this study between the sponsor and the Spanish Agency of Medicines and Medical Devices.

1.12. Study population and total number of patients

The primary objective of the study is to evaluate the safety and toxicology profile of plitidepsin at each dose level administered once a day for 3 consecutive days.

Three cohorts of 9 patients at each of the three dose levels will be included in the study and the recruitment will be sequential in blocks of 3 patients each until a maximum of 27 patients are included in the study.

During the extension period of the study that will start after the recruitment of the 27 patients initially planned an additional 18 patients will be included (6 at each dose level).

1.13. Inclusion and exclusion criteria

1.13.1. Inclusion criteria

1. Patient who agrees to participate in the study by signing the informed consent.
2. Men and women (non-pregnant) aged ≥18 years.
3. PCR-confirmed COVID-19 infection obtained from nasopharyngeal exudate or sample from the lower respiratory tract.
4. Patients who require hospitalisation for COVID-19
5. Onset of symptoms no later than 10 days prior to study inclusion.
6. Men and women with reproductive capacity must agree to use highly effective contraceptive methods (diaphragm plus spermicide or male condom plus spermicide, oral contraceptive combined with a second method of contraceptive implant, injectable contraceptive, permanent intrauterine device, sexual abstinence or vasectomy) during their participation in the study and in the 6 months following the last administration of plitidepsin.
7. In addition, women participating in the study with reproductive capacity must have a negative pregnancy test at enrolment.

1.13.2. Exclusion criteria

1. Patients participating in another clinical trial for COVID-19 infection.
2. Patients receiving treatment with antivirals, interleukin-6 receptor inhibitors and immunomodulatory drugs for COVID-19.
3. Patients who are receiving treatment with chloroquine and derivatives.
4. Evidence of multi-organ failure.
5. Patients requiring support with mechanical ventilation (invasive or non-invasive) at the time of inclusion.
6. D-dimer>4 x LSN.
7. Hb < 9 g/dl.
8. Neutrophils <1000/mm^3.
9. Platelets <100,000/mm^3.
10. Lymphopenia <800/µl.
11. GOT / GPT > 3 X ULN.

12. Bilirubin > 1 X ULN.
13. CPK > 2.5 X ULN.
14. Creatinine clearance <30 ml/min.
15. Troponin elevation > 1.5 x ULN.
16. Clinically relevant heart disease (NYHA> 2).
17. Clinically relevant arrhythmia or previous history/presence of prolonged QT-QTc interval ≥ 450 ms.
18. Pre-existing neuropathies of any type ≥ grade 2.
19. Hypersensitivity to the active substance or to any of the excipients (macrogolglycerol ricinoleate and ethanol)
20. Patients who require or are being treated with strong CYP3A4 inhibitors and inducers (See annex 5).
21. Patients who for any reason should not be included in the study according to the evaluation of the research team.

1.14. Study schedule

Inclusion start date: May 2020

Extension start date: September-October 2020

The study will end with the last follow-up visit of the last patient.

2. CONTENTS

1. PROTOCOL SUMMARY **2**
1.1. SPONSOR IDENTIFICATION 2
1.2. STUDY TITLE 3
1.3. PROTOCOL CODE 3
1.4. DRUG RESEARCH ETHICS COMMITTEE (CEIM) 3
1.5. COMPANY RESPONSIBLE FOR MONITORING 3
1.6. INVESTIGATIONAL PRODUCT 3
1.7. CLINICAL TRIAL PHASE 3
1.8. STUDY OBJECTIVES 3
1.9. STUDY DESIGN 4
1.10. DISEASE UNDER STUDY 6
1.11. ENDPOINTS 6
1.12. STUDY POPULATION AND TOTAL NUMBER OF PATIENTS 7
1.13. INCLUSION AND EXCLUSION CRITERIA 8
1.13.1. Inclusion criteria 8
1.13.2. Exclusion criteria 8
1.14. STUDY SCHEDULE 9
2. CONTENTS **10**
3. ABBREVIATIONS **13**
4. STUDY CHARACTERISTICS **14**
4.1. STUDY IDENTIFICATION 14
4.2. STUDY PHASE 14
4.3. INVESTIGATIONAL PRODUCT DESCRIPTION 14
4.4. SPONSOR INFORMATION 14
4.5. DRUG RESEARCH ETHICS COMMITTEE (CEIM) 15
4.6. COMPANY RESPONSIBLE FOR MONITORING 16
4.7. EXPECTED DURATION OF THE STUDY 16
5. JUSTIFICATION AND OBJECTIVES **16**
5.1. INTRODUCTION 16
5.2. JUSTIFICATION OF THE STUDY 16
5.3. STUDY OBJECTIVES 23
6. STUDY DESIGN **23**
6.1. DESIGN 23
6.2. PRIMARY ENDPOINT 25
6.3. SECONDARY ENDPOINTS 26
7. SCREENING OF PATIENTS **26**
7.1. INCLUSION CRITERIA 27
7.2. EXCLUSION CRITERIA 27
7.3. RANDOMISATION PROCEDURES 28
7.4. MASKING PROCEDURES 28
8. WITHDRAWAL AND DISCONTINUATION CRITERIA **28**
8.1. REASON FOR WITHDRAWAL 28
8.2. MANAGEMENT OF WITHDRAWALS 29
8.3. REPLACEMENT OF SUBJECTS AND RESERVE SUBJECTS 29
9. INVESTIGATIONAL PRODUCT **29**
9.1. DESCRIPTION OF THE INVESTIGATIONAL PRODUCT 29
9.1.1. Description 29
9.1.2. Packaging 29

9.1.3. Shipping, storage and destruction 30
9.2. DOSAGE AND ADMINISTRATION 30
9.3. DURATION OF TREATMENT 31
9.4. CRITERIA FOR DOSAGE MODIFICATION 31
9.5. MANAGEMENT OF INFUSION REACTIONS (E.G. HYPERSENSITIVITY REACTION) 31
9.6. TREATMENT ACCOUNTING AND COMPLIANCE 32
9.7. PROHIBITED DRUGS 32
9.8. DRUG INTERACTIONS 32

10. EVALUATION CALENDAR 35

11. ADVERSE EVENTS 37

11.1. DEFINITIONS 37
11.1.1 Adverse Event (AE) 37
11.1.2 Serious Adverse Event (SAE) 37
11.1.3 Adverse reaction 38
11.2. CHARACTERISTICS OF AN ADVERSE EVENT 38
11.2.1. Severity 38
11.2.2 Determination of causality 38
11.2.3 Expectability 38
11.2.4 Severity 39
11.2.5 Additional considerations 39
11.3. REPORTING PROCEDURES 39
11.3.1. Reporting of Adverse Events (AEs) 39
11.3.2. Reporting Serious Adverse Events (SAEs) 40
11.3.3. Reporting of Serious Adverse Events to the Health Authorities 40
11.3.4. Monitoring of AEs and SAEs 40
11.3.5. AEs or SAEs that occurred after the end of the study 41
11.3.6. Pregnancy and breastfeeding 41
11.3.7. Overdose 42

12. STATISTICAL CONSIDERATIONS 42

12.1. GENERAL CONSIDERATIONS 42
12.2. SAMPLE CALCULATION 42
12.3. ANALYSIS POPULATIONS 43
12.4. ANALYSIS OF THE PRIMARY ENDPOINT 43
12.5. ANALYSIS OF SECONDARY ENDPOINTS 44
12.5.1. Efficacy assessments 44
12.5.2. Secondary safety endpoints 44
12.5.3. Interim safety analysis 45

13. DATA MANAGEMENT AND QUALITY ASSURANCE 45

13.1. CLINICAL TRIAL MONITORING AND AUDITS 45
13.2. AUDITS 45
13.3. INSPECTIONS 46
13.4. REPORTING OF SERIOUS BREACHES 46
13.5. DATA MANAGEMENT 46
13.6. ELECTRONIC CASE REPORT FORMS (ECRF) 47
13.7. WEB-BASED ELECTRONIC CRF 47
13.8. DATA RECORDING IN THE ELECTRONIC CRF 47
13.9. QUERY MANAGEMENT 48
13.10. SOURCE DOCUMENTS 48
13.11. USER IDENTIFIER 48
13.12. CHANGE CONTROL 49
13.13. DOCUMENTATION STORAGE 49

14. ADMINISTRATIVE CONSIDERATIONS 49

14.1. LEGAL CONSIDERATIONS 49
14.2. REVIEW BY THE DRUG RESEARCH ETHICS COMMITTEE 50
14.3. PROTOCOL MODIFICATIONS 50
14.4. INFORMED CONSENT 51

14.5. CONFIDENTIALITY 52
14.6. INSURANCE POLICY 52
14.7. PUBLICATIONS 52

15. ACCEPTANCE OF THE PROTOCOL BY THE SPONSOR 54

16. ACCEPTANCE OF THE PROTOCOL BY THE PRINCIPAL INVESTIGATOR AND COLLABORATORS 55

17. BIBLIOGRAPHIC REFERENCES 56

ANNEX 1. DECLARATION OF HELSINKI 57

ANNEX 2. LIST OF SITES / PRINCIPAL INVESTIGATORS 63

ANNEX 3. SAMPLE PATIENT INFORMATION SHEET AND INFORMED CONSENT FORM 64

ANNEX 4. PREPARATION GUIDE FOR PLITIDEPSIN INFUSION 65

ANNEX 5. CYP3A4 INDUCERS AND INHIBITORS 67

ANNEX 6. NCI-CTCAE CRITERIA 69

ANNEX 7: PLITIDEPSIN SAFETY INFORMATION 70

1. SAFETY 70
1.1.1. Summary of adverse events in completed studies with plitidepsin as a single agent. 70
1.1.2. APL-A-004-98 phase I trial evaluating plitidepsin from Day 1 to Day 5 in patients with solid tumours and low-intermediate grade non-Hodgkin lymphoma 72
2. PHARMACOKINETICS 91
2.1. ADMINISTRATION OF PLITIDEPSIN IN SPECIAL POPULATIONS 93
3. REFERENCES 94

3. ABBREVIATIONS

Abbreviation	Term
AE	Adverse Event
AEMPS	Spanish Agency of Medicines and Medical Devices
AUC	Area Under the Curve
bpm	Beats per minute
bpm	Breaths per minute
CEIm	Drug Research Ethics Committee
CPK	Creatine-phosphokinase
CRF	Case Report Form
EC	European Community
ECG	Electrocardiogram
eCRF	Electronic Case Report Form
eEF1A	Eukaryotic elongation factor
EU	European Union
GCP	Good Clinical Practice
GOT	Glutamic oxaloacetic transaminase
GPT	Glutamate-pyruvate transaminase
HR	Heart rate
IC50	Inhibitory concentration 50
IC90	Inhibitory concentration 90
ICH	International Council for Harmonisation
ICU	Intensive care unit
ID	Identification number
IgE	Immunoglobulin E
IL-6	Interleukin 6
IMV	Invasive mechanical ventilation
ITT	Intention-to-treat
LBR	Lung-blood ratio
LPR	Lung-plasma ratio
N	Nucleocapsid
NIMV	Non-invasive mechanical ventilation
OTI	Orotracheal intubation

Abbreviation	Term
PR	PR interval
Q1	First quartile
Q3	Third quartile
QT	QT interval
RR	Respiratory rate
SAE	Serious Adverse Event
SBP	Systolic blood pressure
WMA	World Medical Association

4. STUDY CHARACTERISTICS

4.1. Study identification

Protocol code:

APLICOV-PC (APL-D-002-20).

Title:

Multicenter, randomized, parallel and proof of concept study to evaluate the safety profile of three doses of Plitidepsin in patients with COVID-19 requiring hospitalization.

4.2. Study phase

Proof of concept.

4.3. Investigational product description

Plitidepsin.

4.4. Sponsor information

Pharma Mar, SA

Contact Person:

Dr Maria José Pontes Valero
Associate International Medical Affairs Director
E-mail[redacted]
Tel:[redacted]
Fax:[redacted]

Dr María-Angeles Forés,
International Medical Affairs Director
e-mail[redacted]
Tel:[redacted]

Postal address:

Avda. de los Reyes, 1.
Polígono Industrial “La Mina”.
28770 Colmenar Viejo.
Madrid, Spain.

Pharmacovigilance contact person:

Hervé Dhellot
EEA Qualified Person for Pharmacovigilance and Senior Pharmacovigilance Manager
E-mail[redacted]
Tel (24 hours)[redacted]
Tel[redacted]
Fax:[redacted]

Postal address:

Avda. de los Reyes, 1.
Polígono Industrial “La Mina”.
28770 Colmenar Viejo.
Madrid, Spain.

Coordinating investigators:

Dr Vicente Estrada
Hospital Clínico San Carlos
Department of Infectious Diseases

Dr Jesús Fortún Abete
Hospital Ramon y Cajal
Department of Infectious Diseases

Dr José Barberán
Hospital HM Montepríncipe,
Department of Internal Medicine

4.5. Drug Research Ethics Committee (CEIm)

Drug Research Ethics Committee of HM Hospitales

4.6.Company responsible for monitoring

APICES

Av. Antonio López, 16 - 1ºA

28320 Pinto (MADRID)

Tel:[redacted]

Fax:[redacted]

E-mail[redacted]

4.7.Expected duration of the study

Inclusion start date: May 2020

Extension start date: September-October 2020

The study will end with the last follow-up visit of the last patient.

5. JUSTIFICATION AND OBJECTIVES

5.1.Introduction

Last December, the World Health Organisation received information on a group of cases of pneumonia of unknown aetiology. The cause of this pneumonia was identified as a new virus in the Coronaviridae family (SARS-CoV-2) and the clinical picture associated with the virus has been named COVID-19.

In January 2020 the first case was reported in Spain and in mid-February the first patient died of it. The growth of confirmed cases has been exponential since then, with the Community of Madrid being the most affected region.

As of April 22, the Coordination Centre for Health Alerts and Emergencies of the Ministry of Health has reported that in Spain there have been 208,389 cases, 21,717 deaths and 85,915 patients cured. The Autonomous Communities with the highest cumulative incidence in the last 14 days are La Rioja, Madrid, Castilla León and Castilla-La Mancha.

COVID-19 is currently a public health emergency. The onset of a virus unknown until now has made it necessary to take measures taking into account the existing scientific knowledge with similar viruses and past situations.

5.2.Justification of the study

Mortality associated with COVID-19 disease appears to be associated with a) severe respiratory failure secondary to respiratory distress and b) an

inflammatory status caused by a cytokine storm. Thus, the proportion of patients with severe disease requiring hospitalisation with or without high-flow oxygen supplements and patients requiring mechanical ventilatory support was estimated to be close to 15% and 5%, respectively, in the initial series from China. However, in our setting, the figures reported by the health authorities are much higher, reaching 30% of serious cases requiring hospitalisation in the city of Madrid without the need for mechanical ventilation and close to 10% of patients requiring mechanical ventilation. Likewise, the duration of the need for mechanical ventilation in the Chinese series is much shorter than that reported in cities such as Madrid, so the usual flow of patients to intensive care units is being altered by the prolonged stay of patients. This is putting an enormous burden on hospital services, which has made it necessary to take extraordinary, unprecedented measures. In any case, it is not known whether the magnitude of the complications initially described can be avoided or reduced through the use of antivirals in patients with early-stage COVID-19 pneumonia, since once the cytokine storm and respiratory distress take place, it is very difficult for an antiviral drug to have a beneficial therapeutic effect.

Plitidepsin is a cyclic depsipeptide originally isolated from a Mediterranean marine tunicate (*Aplidium albicans*) and currently manufactured by full chemical synthesis. It is licensed and marketed in Australia under the brand name plitidepsin for the treatment of multiple myeloma. It belongs to the family of didemnins, discovered in the 1980s, which have a powerful capacity against DNA and RNA viruses [1,2].

In eukaryotic cells, plitidepsin has been unequivocally shown to target the eukaryotic elongation factor (eEF1A) [3], which has a key role in modulating interaction with other proteins [4], some of which may be essential in the replication of various viruses. It is noteworthy that one of the aforementioned proteins is the coronavirus N protein, which is produced abundantly within infected cells and is known to interact with elongation factor EF1A [5]. The interaction between plitidepsin and EF1A could therefore reduce the efficacy of de novo viral capsid synthesis and consequently entail a decrease in viral load.

With these arguments, the in vitro antiviral activity of plitidepsin in a human hepatoma cell line (Huh-7) infected with the HCoV-229E-GFP virus was studied [6,7]. In this sense, it must be highlighted that the N protein of this coronavirus has a protein homology of more than 90% with the SARS-CoV-2 homologous protein and, although it has not been described, all coronaviruses need their N (nucleocapsid) protein to bind to EF1A in order to replicate effectively and synthesise viral proteins. No binding of N to EF1A means no viability for the spread of the virus. For a short while, confluent cultures of Huh-7 were infected at a multiplicity of infection (MOI) of 0.01 pfu/cell, with a viral inoculum of $3x10^7$ pfu/ml and after 8 hours, plitidepsin was added at concentrations ranging from 0.5 nM to 50 µM. The cultures with plitidepsin were incubated for 48 hours and

then viral viability was measured by fluorescence. The results obtained showed an antiviral effect induced by plitidepsin at concentrations as low as 0.5 nM (0.555 µg/l), much lower than those reported with other antivirals [8].

Estimate of target concentrations in vitro.

Assuming that the IC50 of the aforementioned in vitro experiment (0.5 nM or 0.555 µg/l) and the unbound fraction of plitidepsin in human plasma (f_u) is 1%, and using equation 2 (assuming a Hill slope of 1), IC90 has been estimated at 4.995 µg/l.

Eq.1

$$E = \frac{E_{max} \cdot C_{total} \cdot f_u}{(IC50 + C_{total} \cdot f_u)}$$

Eq.2

$$IC50_{in\ vitro} = \frac{E_{max} \cdot C_{total} \cdot f_u - E \cdot C_{total} \cdot f_u}{E}$$

Since the cells of the experiment were cultured in a medium with 10% bovine serum, an unbound fraction above 0.96 was estimated. Therefore, to ensure active plitidepsin concentrations in tissues (e.g. lung) with high tropism of coronavirus, the concentration should be ≥ 0.555 µg/l.

Selection of the dosage regimen for the treatment of SARS-CoV-2 infection.

For the estimation of the total target plasma concentration of plitidepsin associated with the concentration in lungs above IC50 (≥ 0.555 µg/l), the lung-plasma ratio and the unbound fraction of plitidepsin obtained were taken into account in the studies below.

Tissue distribution data

In an in vivo distribution study in rats [9], in which an intravenous bolus dose (0.2 mg/kg) of ^{14}C-plitidepsin was administered, a significant increase in radioactivity was observed in the lungs of animals. This preferential distribution indicates a lung-blood (LBR) and lung-plasma (LPR) AUC ratio of around 8- and 543-fold, respectively.

Plasma protein binding

In human and rat plasma, plasma protein binding of plitidepsin was independent of drug concentration (100, 200 and 500 ng/ml), estimated at 98% and 96%, respectively [10].

Estimation of the total plasma concentrations associated with the target concentrations (IC50, IC90 and 3xIC90) in vitro.

The total plasma concentration (µg/l) of plitidepsin associated with a lung exposure above the in vitro target concentration (IC50 = 0.555 µg/l) was estimated at 0.05 µg/l, by means of equation 3 (see below), assuming that 1) SARS-CoV-2 $IC50_{total,\ in\ vitro}$ is similar to that described for HCoV-229E, and 2) the lung distribution in humans is similar to that observed in rats.

Eq.3

$$\mathrm{IC50}_{\mathrm{total,plasma}} = \frac{IC50_{total,\ in\, vitro}}{f_{u,human} \cdot LBR_{rat}}$$

where $IC50_{total\ plasma}$ is the total target plasma concentration (µg/l), $IC50_{total\ in\ vitro}$ is the concentration used in the in vitro experiment (µg/l), $f_{u,\ human}$ is the unbound fraction in human plasma and LBR_{rat} is the lung-blood ratio in the distribution study in rats.

The total plasma concentration (µg/l) of plitidepsin associated with lung exposure above the target IC90 in vitro concentration (IC90 = 4.995 µg/l) was estimated to be 0.46 µg/l, using the equation above. Therefore, total plasma plitidepsin concentrations above 1.38 µg/l would be associated with a lung exposure 3 times higher than the target IC90 in vitro concentration.

Phase 1 studies with plitidepsin

In order to define the dosage and regimen of plitidepsin as a single agent, an extensive phase 1 clinical development programme was carried out, which explored five administration schedules, including a total of 215 adult patients with various types of tumours, both solid and haematological. Table 1 shows the dosing schedules tested during phase 1, as well as the number of patients, the maximum tolerated dose, the recommended dose and the intensity of the weekly dose.

Table 1: Phase 1 studies of plitidepsin as a single agent.

	24-hour D1,8,15 q4wk	3-hour D1,15 q4wk	1-hour D1,8,15 q4wk	24-hour D1,15 q4wk		1-hour iv D1-5 q3wk
				Plitidepsin	Plitidepsin + L-carnitine	
**No. of patients**	35	27	48	67	20	37
**MTD (mg/m^2)**	4.50	6	3.60	6	8	1.35
**RD (mg/m^2)**	3.75	5	3.2	5	7	1.2
**DI at RD (mg/m^2/week)**	2.8	2.5	2.4	2.5	3.5	2.0

D, day; MTD, maximum tolerated dose; RD: recommended dose; DI: Dose intensity; iv, intravenous.

Figure 1 shows these phase 1 studies together with phase 2 and 3 studies in the same dosing schedule, in which a pharmacokinetic sub-study was also

performed.

Figure 1: Clinical studies with pharmacokinetic sub-study, sorted by dosing schedule and phase.

Regimen	Phase I escalation studies: Single agent	Phase I escalation studies: Combination	Phase II studies: Solid	Phase II studies: Hematologic	Phase III
24h D1, 8, 15 q4wk	APL-A-001a-98 n=35; 97% w/PK				
3h D1, 15 q4wk	APL-A-001b-98 n=27; 96% w/PK	APL-A-011-08 w/DCT or BVZ n=16; 81% w/PK<br>APL-A-012-13 w/bortezomib n=22; 86% w/PK	APL-B-002-02 M. thyroid ca. n=16; 100% w/PK<br>APL-B-005-02 Urothelium ca. n=21; 90% w/PK<br>APL-B-007-02 Melanoma n=37; 100% w/PK<br>APL-B-011-02 Prostate ca. n=8; 100% w/PK	APL-B-014-03 r/r MM w/ DTX n=51; 88% w/PK	APL-C-001-09 r/r MM w/DTX *vs.* DTX n=204; 68% w/PK
1h D1, 8, 15 q4wk	APL-A-002-98 n=49; 94% w/PK	APL-A-006-05 w/cisplatin n=20; 100% w/PK<br>APL-A-010-08 w/GZB or SRF n=44; 73% w/PK	APL-B-006-02 SCLC n=19; 79% w/PK<br>APL-B-016-05 Melanoma w/DTIC n=84; 80% w/PK	APL-B-013-02 NHL n=34; 71% w/PK<br>APL-B-015-04 ALL n=17; 100% w/PK	
24h D1,15 q4wk	APL-A-003-98 n=67; 66% w/PK		APL-B-001-02 RCC and CRC n=81; 53% w/PK		
1h D1-5 q3wk	APL-A-004-98 n=37; 100% w/PK				

n: number of patients treated with plitidepsin; **% w/PK**: percentage of treated patients who participated in the pharmacokinetic sub-study.

Daily schedule (D1-5 every three weeks)

Among all the dosing schedules explored, the one used in study APL-A-004-98 [11] in 37 patients with solid tumours is considered the ideal one for a non-oncological population, maintaining sustained exposure and allowing daily adjustments of treatment based on the patient's response. At the recommended dose for cancer patients, 1.2 mg/m^2 (equivalent to a flat dose of 2.2 mg), this plitidepsin schedule showed a favourable safety profile compared to other oncological agents.

Dose selection

A validated pharmacokinetic population model of plitidepsin [12] was used to identify at what doses the total plasma concentrations would reach the

estimated target concentrations of antiviral activity set at 0.05 µg/l (IC50), 0.46 µg/l (IC90) and 1.38 µg/l (3xIC90) (see above).

In the pharmacokinetic population model of plitidepsin, a link between body surface area (BSA) (1.2-2.5 m²) and plasma clearance of plitidepsin was not observed, therefore flat dosing is considered preferable in order to facilitate the administration of the compound at the sites.

Plitidepsin is preferentially distributed in the erythrocyte, resulting in a blood/plasma ratio of between 3.37 and 4.45.

It should be noted that the half-life value of plitidepsin of 6.7 days corresponds to the pharmacokinetic population analysis performed with concentrations of whole blood and not plasma. Quantifications in whole blood allow detectable concentrations of plitidepsin to be obtained several days after administration, resulting in a long elimination phase and a long half-life. However, during this elimination phase, plasma concentrations, which are effective from the point of view of activity, safety and potential interactions, are undetectable.

As proof of this, the half-life calculated with non-compartmental analysis based on plasma concentrations (study APL-B-015-14) is 48.1 hours.

Figure 2 illustrates the simulation of the total plasma plitidepsin concentration profiles vs. time after a daily dose (D1-D3) of 1.5 mg, 2.0 mg and 2.5 mg.

Figure 2: Total plasma concentration profiles vs. plitidepsin time predicted for the proposed dosing schedule and administration.

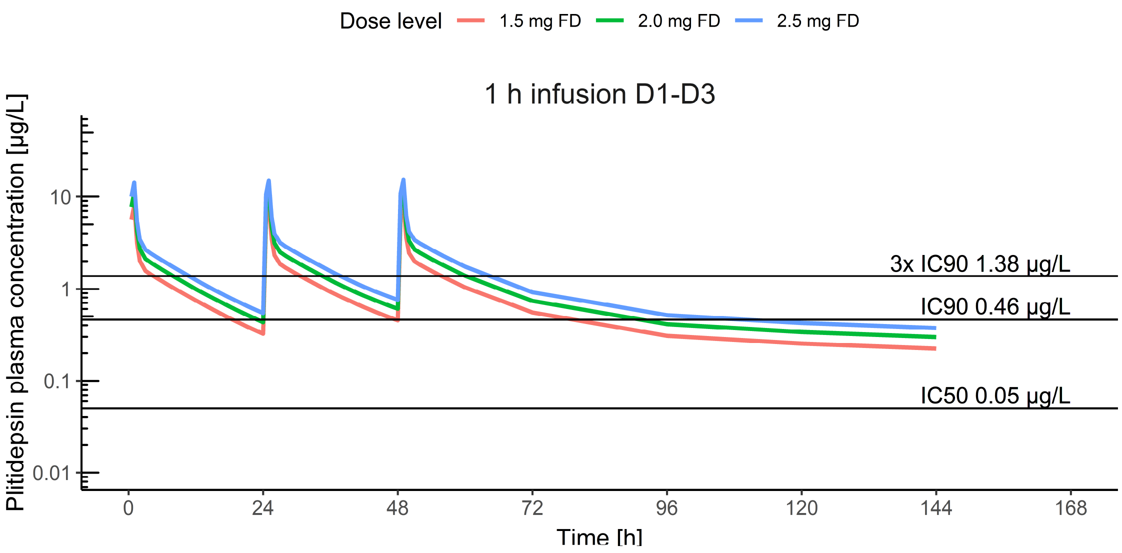

The horizontal black lines represent the total plasma concentrations associated with concentrations in lungs equivalent to IC50, IC90 and 3xIC90 in vitro.

With all three dosage levels (1.5 mg, 2.0 mg and 2.5 mg), plasma concentrations above IC50 would be obtained throughout the treatment period and would remain above IC90 during most of the administration interval. Accumulation after three repeated administrations is minimal.

The cumulative doses associated with the proposed administration schedules (7.5 mg, 6.0 mg and 4.5 mg) are approximately 70%, 55% and 40% of that defined in the phase I study APL-A-004-98 (8.4-12.0 mg), recommended for patients with solid tumours based on safety criteria. **Phase I study of plitidepsin (APL-A-004-98)**

In the phase I study (APL-A-004-98) 37 patients were treated with plitidepsin once daily with a 1-hour infusion for 5 days at different dose levels.

Patients were included in 10 cohorts at different doses from 0.08 mg/m^2 to 1.5 mg/m^2, on days 1 to 5, with the maximum tolerated dose being 1.35 mg/m^2.

Since fixed doses of plitidepsin are to be administered in the proposed study, an analysis of the adverse events observed has been conducted in study APL-A-004-98 in 19 patients treated at total doses of 0.5 mg to 2.5 mg separated into 5 groups.

1) 0.5 mg dose level (± 0.2 mg). Total dose for 5 days 2.5 mg. N = 3
2) 1.0 mg dose level (± 0.2 mg). Total dose for 5 days 5.0 mg. N = 5
3) 1.5 mg dose level (± 0.2 mg). Total dose for 5 days 7.5 mg. N = 4
4) 2.0 mg dose level (± 0.2 mg). Total dose for 5 days 10.0 mg. N = 3
5) 2.5 mg dose level (± 0.2 mg). Total dose for 5 days 12.5 mg. N = 4

The proposed doses for the clinical trial of plitidepsin in COVID 19 would be 1.5 (3-day total dose of 4.5 mg), 2 mg for 3 days (3-day total dose of 6 mg) and 2.5 mg during 3 days (7.5 mg total 3-day dose), bringing dose levels between dose level 1 and 3 of the phase I study (APL-A-004-98).

Taking into account that the safety data available today comes from patients with solid tumours previously treated with chemotherapy and treated with an administration regimen of plitidepsin for 5 consecutive days, we propose a multicentre, uncontrolled, proof-of-concept clinical trial, with three treatment arms to evaluate the safety profile of an administration scheme over three consecutive days at three different dose levels of plitidepsin, in adult patients with a confirmed diagnosis of COVID-19 who require hospitalisation.

Additional safety information for plitidepsin is included in Annex 7.

5.3. Study objectives

Primary objective:

To determine the safety and toxicology profile of plitidepsin at each dose level administered in patients admitted for COVID-19 according to the proposed administration scheme.

Secondary objectives:

- To assess the efficacy of plitidepsin in patients with COVID-19 at the proposed dose levels by:
  - Change in SARS-CoV-2 viral load from baseline
  - Time until negative detection of SARS-CoV-2 by PCR
  - Cumulative incidence of disease severity. The evaluation will be based on:
    - Mortality
    - Need for invasive mechanical ventilation and/or ICU admission
    - Need for non-invasive mechanical ventilation
    - Need for oxygen therapy
- Selection of the recommended dose levels of plitidepsin for a phase II/III efficacy study

6. STUDY DESIGN

6.1. Design

Multicentre, randomised clinical trial with three treatment arms.

Three cohorts of 9 patients will be included in the study. Each of the cohorts of 9 patients will include 3 arms (A, B, and C) at different dose levels with 3 patients in each arm, up to a maximum of 27 patients evaluable for safety.

The established dosage levels are as follows:

- Arm A) 1.5 mg of plitidepsin administered as a 1.5-hour infusion, once a day for 3 consecutive days.
- Arm B) 2.0 mg of plitidepsin administered as a 1.5-hour infusion, once a day for 3 consecutive days.
- Arm C) 2.5 mg of plitidepsin administered as a 1.5-hour infusion, once a day for 3 consecutive days.

ARM	Dosage level	Daily dose (mg)	Total dose (mg)
Arm A	1	1.5 mg x 3 days	4.5 mg
Arm B	2	2.0 mg x 3 days	6.0 mg
Arm C	3	2.5 mg x 3 days	7.5 mg

Recruitment will be sequential in each of the three arms. The first cohort of 3 patients in arms B and C (2.0 mg and 2.5 mg) will not open until the data have been analysed for the first 3 patients at the dose level immediately below (arms A or B) without observing any unacceptable toxicity, taking into account the data of the three patients included in each arm with a 12-day follow-up from the end of treatment (day +15 from the start of treatment).

When patients are to be included at more than one dose level (arm), patients will be randomised in a 1:1 or 1:1:1 ratio as appropriate.

The following criteria have been established to continue the inclusion of patients in the different arms:

- If 0-1 of the first three patients treated with the dose of 1.5 mg x 3 days experience any adverse event classed as grade 3 or higher within twelve days after administration of the last dose, three additional patients will be enrolled in the 1.5 mg arm and recruitment of the first three patients in the 2.0 mg arm will start. Recruitment in the 2.5 mg arm will be opened after it has been proven that only 0-1 of the three patients in the first cohort in the 2.0 mg arm have grade 3 or greater toxicity within twelve days of follow-up after administration of the last dose of 2.0 mg.
-
- If 2 or more patients in any of the arms present an adverse event classed as grade 3 or higher, the sponsor will interrupt the recruitment of new patients and will jointly assess with the Spanish Agency of Medicines and Medical Devices whether or not to continue recruitment in that arm and in arms with a higher dose.

Intra-patient dose change/increase is not allowed.

Once the 27 patients (9 at each dose level) contemplated in the study protocol have been included, an extension period will be opened to include 18 additional patients (6 at each plitidepsin dose level) to extend the collection of safety information on the different doses and additionally to collect efficacy data. After this extension period, data will be available for 15 patients at each plitidepsin dose level.

During the extension period, patients will be randomised in a 1:1:1 ratio as appropriate to each dose level (arm).

During the extension period of the study, if in any of the arms, 2 or more patients present an adverse event classed as grade 3 or higher related to plitidepsin, the sponsor will interrupt the recruitment of new patients at that dose level and higher, and will assess jointly with the Spanish Agency of Medicines and Medical Devises the convenience of continuing recruitment in that arm and in arms with a higher dose. Additionally, the sponsor will forward weekly follow up records to the Spanish Agency of Medicines and Medical Devices, with the information of all the adverse event classed as grade 3 or higher, reported, whether or not related to plitidepsin, for review and joint assessment if necessary.

After the joint evaluation of the safety and efficacy data obtained in the present study between the sponsor and the Spanish Agency of Medicines and Medical Devices, the recommended dose level will be selected to evaluate the efficacy of the treatment in patients with COVID-19 in a subsequent randomised phase II/III study.

The final statistical analysis that will be the basis of the final report of the study will be done on the total population of 45 patients once the extension of the study has been completed The data of the first 27 patients will be analysed in an intermediate statistical analysis.

6.2.Primary endpoint

<u>Primary endpoint:</u>

To determine the safety and toxicological profile of plitidepsin at each dose level administered in patients admitted for COVID-19, the following endpoints will be analysed:

- Frequency of the following ≥ grade 3 adverse events according to the NCI-CTCAE criteria v 5.0 at 3, 7, 15 and 31 days:
  - Haematological
    - Neutropenia
    - Thrombopenia
    - Anaemia
    - Lymphopenia
  - Non-haematological
    - CPK increase
    - ALT and/or AST increase
    - Total bilirubin or direct bilirubin increase
    - Neurotoxicity

    - QT-QTc interval prolongation
  - Other ≥ grade 3 adverse events

- Percentage of patients in which treatment cannot be completed and reasons.
- Percentage of patients with AEs and SAEs at days 3, 7, 15 and 31.
- Changes from baseline (day -1 or day 1 before administration of the study drug) in haematological and non-haematological parameters on days 3, 7, 15 and 31.
- Percentage of patients with ECG abnormalities on days 2, 3, 4, 5, 6, 7, 15 and 31.

6.3.Secondary endpoints

- Change in SARS-CoV-2 viral load from baseline (day -1 or day 1 before administration of the study drug) measured at days , 4, 7, 15 and 31.
- Time until the detection of COVID-19 by PCR obtained from nasopharyngeal exudate or sample from the lower respiratory tract compared to baseline (day -2 or -1 or day 1 before administration of the study drug).
- Mortality at 7, 15 and 31 days.
- Percentage of patients requiring invasive mechanical ventilation and/or ICU admission at 7, 15 and 31 days.
- Percentage of patients requiring non-invasive mechanical ventilation at 7, 15 and 31 days.
- Percentage of patients requiring oxygen therapy at 7, 15 and 31 days.
- The recommended dose level will be selected after the joint evaluation of the safety and efficacy data obtained in this study between the sponsor and the Spanish Agency of Medicines and Medical Devices.

7. SCREENING OF PATIENTS

When a patient is considered eligible to participate in the study, they will be offered to participate in it and receive the Patient Information Leaflet along with all additional information required to obtain informed consent.

7.1. Inclusion criteria

1. Patient who agrees to participate in the study by signing the informed consent.
2. Men and women (non-pregnant) aged ≥18 years.
3. PCR-confirmed COVID-19 infection obtained from nasopharyngeal exudate or sample from the lower respiratory tract.
4. Patients who require hospitalisation for COVID-19
5. Onset of symptoms no later than 10 days prior to study inclusion.
6. Men and women with reproductive capacity must agree to use highly effective contraceptive methods (diaphragm plus spermicide or male condom plus spermicide, oral contraceptive combined with a second method of contraceptive implant, injectable contraceptive, permanent intrauterine device, sexual abstinence or vasectomy) during their participation in the study and in the 6 months following the last administration of plitidepsin.
7. In addition, women participating in the study with reproductive capacity must have a negative pregnancy test at enrolment.

7.2. Exclusion criteria

1. Patients participating in another clinical trial for COVID-19 infection.
2. Patients receiving treatment with antivirals, interleukin-6 receptor inhibitors and immunomodulatory drugs for COVID-19.
3. Patients who are receiving treatment with chloroquine and derivatives.
4. Evidence of multi-organ failure.
5. Patients requiring support with mechanical ventilation (invasive or non-invasive) at the time of inclusion.
6. D-dimer> 4 x LSN.
7. Hb < 9 g/dl.
8. Neutrophils <1000/mm^3.
9. Platelets <100,000/mm^3.
10. Lymphopenia <800/µl.
11. GOT / GPT > 3 X ULN.
12. Bilirubin > 1 X ULN.
13. CPK > 2.5 X ULN.

14. Creatinine clearance <30 ml/min.
15. Troponin elevation > 1.5 x ULN.
16. Clinically relevant heart disease (NYHA> 2).
17. Clinically relevant arrhythmia or previous history/presence of prolonged QT-QTc interval ≥ 450 ms.
18. Pre-existing neuropathies of any type ≥ grade 2.
19. Hypersensitivity to the active substance or to any of the excipients (macrogolglycerol ricinoleate and ethanol)
20. Patients who require or are being treated with strong CYP3A4 inhibitors and inducers (See annex 5).
21. Patients who for any reason should not be included in the study according to the evaluation of the research team.

7.3. Randomisation procedures

Patients will be randomised, provided patients are included at more than one dose level (arm), in a 1:1 or 1:1:1 ratio as appropriate, once the investigator has confirmed that they meet the selection criteria and the patient has given informed consent.

During the extension period, patients will be randomised in a 1:1:1 ratio as appropriate to each dose level (arm).

The randomisation will be done in blocks.

Randomisation will be done through registration in the study's eCRF. The system will assign a unique randomisation number for each patient. This number should be included in all study documents related to the patient.

7.4. Masking procedures

Not applicable.

8. WITHDRAWAL AND DISCONTINUATION CRITERIA

8.1. Reason for withdrawal

Patients will be excluded or withdrawn from the study for any of the following reasons:

- Withdrawal of consent by the patient.
- Administration of treatments not allowed in the protocol.

All cases of a patient's withdrawal from the study must be documented in the Case Report Form.

8.2. Management of withdrawals

Patients who receive at least one dose of plitidepsin and withdraw from the study will be followed for at least 28 days after administration of the last dose of plitidepsin to follow up on possible open adverse events at withdrawal.

8.3. Replacement of subjects and reserve subjects

Patients who sign the study consent and are not randomised will be replaced.

Patients who discontinue study treatment before the end of the three proposed administrations for reasons other than ≥ grade 3 adverse events will be replaced.

9. INVESTIGATIONAL PRODUCT

9.1. Description of the investigational product

Plitidepsin is licensed in Australia for the treatment of multiple myeloma.

Safety information for plitidepsin is included in Annex 7.

9.1.1. Description

Plitidepsin is supplied as a powder for concentrate for solution for infusion at a concentration of 2 mg/vial. Before use, the vials are reconstituted with 4 ml of reconstitution solution to obtain a colourless to slightly yellowish solution containing 0.5 mg/ml of plitidepsin, 25 mg/ml of mannitol, 0.15 ml/ml of macrogolglycerol ricinoleate oil, 0.15 ml/ml of ethanol and 0.70 ml/ml of water for injection. An additional dilution should be made in any suitable intravenous solution prior to infusion.

9.1.2. Packaging

Plitidepsin 2 mg is supplied in a Type I clear glass vial with a bromobutyl rubber stopper covered with an aluminium seal. Each vial contains 2 mg of plitidepsin.

The solvent for the reconstitution of macrogolglycerol ricinoleate (polyoxyl 35 castor oil)/absolute ethanol/water for injection, 15%/15%/70% (v/v/v) is supplied in a Type I colourless glass vial. The ampoules have a volume of 4 ml.

Plitidepsin will be labelled with the study protocol code, the batch number, the content, the expiry date, the storage conditions, the name of the investigator and the sponsor. The study drug will be labelled in accordance with Annex 13 of the European Good Manufacturing Practices.

9.1.3. Shipping, storage and destruction

Plitidepsin should be stored between 2ºC and 8ºC and the vials should be kept in the outer carton to protect them from light.

The drug in these conditions is stable for 60 months.

Any unused product or waste materials must be disposed of in accordance with local requirements for cytotoxic drugs.

9.2. Dosage and administration

Patients included in the study will be randomised in a 1:1:1 ratio to receive:

- Arm A) 1.5 mg of plitidepsin administered as a 1.5-hour infusion, once a day for 3 consecutive days.
- Arm B) 2.0 mg of plitidepsin administered as a 1.5-hour infusion, once a day for 3 consecutive days.
- Arm C) 2.5 mg of plitidepsin administered as a 1.5-hour infusion, once a day for 3 consecutive days.

See guidelines for medication preparation in Annex 4.

Personnel should be trained and qualified to reconstitute and dilute the medication and should wear protective clothing including a mask, goggles and gloves during reconstitution and dilution.

Accidental contact with skin, eyes or mucous membranes should be treated immediately with large amounts of water.

All patients should receive the following prophylactic medications 20-30 minutes before the infusion of plitidepsin:

- Diphenhydramine hydrochloride 25 mg iv or equivalent.
- Ranitidine 50 mg iv or equivalent.
- Dexamethasone 8 mg intravenous.
- Ondansetron 8 mg i.v. slow infusion 15 minutes or equivalent

Ondansetron 4 mg oral will be administered every 12 hours until 48 hours after the last administration of plitidepsin.

After reconstitution of the 2 mg plitidepsin vial with 4 ml of the solution for reconstitution of macrogolglycerol ricinoleate/ethanol/water for injection, the reconstituted solution should be diluted and used immediately after preparation.

If not used immediately, storage times and conditions until use are the responsibility of the user.

The reconstituted concentrated solution of the drug product has been shown to be physically, chemically and microbiologically stable for 24 hours under refrigerated conditions (5ºC±3ºC) and for 6 hours when stored in the original vial under indoor light at room temperature.

If storage is required before administration, solutions should be stored refrigerated and protected from light and should be used within 24 hours after reconstitution.

9.3. Duration of treatment

Patients included of the study will receive treatment for 3 days.

9.4. Criteria for dosage modification

No dose reduction has been contemplated.

9.5. Management of infusion reactions (e.g. *hypersensitivity reaction*)

In the event of a serious/life-threatening infusion reaction (grade ≥ 3), the plitidepsin infusion should be discontinued and immediate treatment with oxygen and bronchodilators should be considered. Treatment should be administered with diphenhydramine iv 50 mg or equivalent, hydrocortisone iv 100 mg and epinephrine (adrenaline) should be added if clinically indicated. The infusion should not be resumed and treatment with plitidepsin should be discontinued.

For mild or moderate/non-life threatening reactions (grade ≤ 2) such as allergic symptoms, the infusion should be stopped immediately and vital signs and pulse oximetry will be continuously monitored. Before resuming infusion of plitidepsin, iv dexamethasone 4 mg premedication (or equivalent) must be administered 20-30 minutes beforehand. If symptoms persist after stopping the infusion and administering dexamethasone, diphenhydramine 50 mg iv or equivalent and hydrocortisone 100 mg bolus iv or equivalent will be administered.

Symptoms and vital signs should be re-evaluated after 30 min. If they are normal or have improved, the infusion may be resumed at a third of the initial infusion rate during the first hour. Signs and symptoms should be continuously monitored during the infusion. Subsequently, the infusion rate could be increased according to tolerance. Any additional infusion will be started at a reduced infusion rate and IV prophylactic premedication will be administered as described above. The patient will be monitored during the first hour after the infusion to detect symptoms. If no hypersensitivity reactions are observed, the

infusion rate could be increased again. In any case, if symptoms do not improve after 30 minutes, anti-H1 and/or corticosteroids will be administered until resolution, and treatment with plitidepsin will be discontinued.

9.6. Treatment accounting and compliance

Study medication should be stored in a safe place with limited access, under the conditions established in the investigator's brochure. The medication will be labelled with the study protocol code, batch number, content, expiry date, storage conditions, investigator and name of the sponsor.

The monitor will supervise the study medication in the hospital pharmacy prior to destruction, after accounting for the medication has been completed. Medication accounting will be documented and amounts shipped, dispensed, returned and destroyed will be verified.

All unused medication provided by the sponsor will be destroyed at each participating site and properly documented. If requested by the sponsor, unused medication will be returned to the sponsor.

All study drug inventory forms should be available for inspection by authorised representatives of the sponsor or by health authority inspectors. The investigator is responsible for counting all used and unused study supplies at their site.

9.7. Prohibited drugs

Concomitant administration of interleukin-6 receptor inhibitors and immunomodulatory drugs is not permitted.

Concomitant use of chloroquine and derivatives is not permitted.

Patients may receive other pharmacological treatments for COVID-19 at the discretion of the investigator after 24 hours from the administration of the last dose of plitidepsin.

9.8. Drug interactions

There have been no clinical studies on drug interactions.

If the patient is already under treatment with chloroquine or one of its derivatives and given its arrhythmogenic potential, the patient may not be included in the study.

Effect of other medicinal products on plitidepsin

Interactions with CYP3A4 inhibitors and inducers

In vitro studies indicate that CYP3A4 is the major enzyme involved in plitidepsin metabolism, followed by CYP2A6 and CYP2E1. However, a pharmacokinetic population analysis of plitidepsin including 283 patients indicated that concomitant administration of CYP3A4* inhibitors and inducers does not affect exposure to plitidepsin [12]. However, it cannot be ruled out that concomitant treatments that inhibit or induce CYP3A4 may modify the efficacy and/or increase the probability of side effects associated with plitidepsin, so they should be avoided.

- Co-administration with CYP3A4 inhibitors and inducers should be avoided, as they may affect the plasma concentration of plitidepsin.
- Co-administration with CYP3A4 inhibitors and moderated inducers should be used with caution, as an effect on plitidepsin exposure cannot be excluded.

Interactions with CYP3A4 inhibitors

Plitidepsin should not be administered with potent inhibitors CYP3A4 (e.g. grapefruit juice, clarithromycin, itraconazole, nefazodone, telithromycin, voriconazole). Potent inhibitors CYP3A4 should be discontinued before starting treatment and during treatment with plitidepsin. They may be re-administered 24 hours after the last dose of plitidepsin.

Moderate inhibitors CYP3A4 (e.g., aprepitant, diltiazem, erythromycin, fluconazole, verapamil) should be used with caution, as increased exposure to plitidepsin cannot be excluded

Interactions with CYP3A4 inducers

To avoid a decrease in the effectiveness of plitidepsin, plitidepsin should not be administrated with potent inducers of CYP3A4 enzyme, such as anticonvulsants (phenytoin, phenobarbital or carbamazepine), rifampicin, rifabutin and St. John's wort, unless there are no therapeutic alternatives. The appropriate initial dose for patients taking potent inducers has not been defined. They may be administered again 24 hours after the last dose of plitidepsin.

CYP3A4 moderate inducers (e.g. bosetan, modafinil, nafcillin) should be used with caution, as a reduction of exposure to plitidepsin cannot be excluded.

A list of CYP3A4 inducers and inhibitors is included in Annex 5.

Effect of plitidepsin on other drugs

In vitro studies did not show a potential for plitidepsin to inhibit or induce metabolism of other drugs. However, the results for ruling out the potential inducing effect of plitidepsin on the CYP2B6 enzyme were inconclusive. The potential effect of plitidepsin on CYP2B6 has not been studied in vivo.

Therefore, decreases in plasma concentrations of drugs that are substrates of this enzyme cannot be ruled out when co-administered with plitidepsin.

10. EVALUATION CALENDAR

	**Baseline**	**Day 1**	**Day 2**	**Day 3**	**Day 4**	**Day 5**	**Day 6**	**Day 7**	**Day 15 (± 1 day)**	**Day 31 (± 3 days)[14]**
Informed consent[1]	X									
Selection criteria[2]	X									
Medical record	X									
Vital signs[3]	X	X	X	X	X	X	X	X	X	X
Demographics	X									
Physical examination	X									
Electrocardiogram[4]	X	X	X	X	X	X	X	X	X	X
Complete blood count[5]	X	X*	X	X	X	X	X	X	X	X
Biochemistry[6]	X	X*	X	X	X	X	X	X	X	X
Coagulation[7]	X	X*	X	X	X	X	X	X	X	X
C-reactive protein[8]	X	X*	X	X	X	X	X	X	X	X
Viral load[15]	X				X			X	X	X
PCR COVID-19[9]	X				X			X	X	X
Chest x-ray[10]	X	Based on clinical criteria								
Pregnancy test[11]	X									
Treatment		X	X	X						
Other pharmacological treatments for COVID-19[12]			X	X	X	X	X	X	X	X
Adverse events[13]	X	X	X	X	X	X	X	X	X	X

	* It will not be necessary to repeat the tests on day 1 if they were carried out in the 24 hours prior to administration of plitidepsin.<br>1 The patient will sign the informed consent before undergoing any specific study procedure that is not part of normal clinical practice.<br><br>2 The patient must meet all the inclusion criteria and none of the exclusion criteria in order to be included in the study.<br><br>3 Vital signs will be measured **every 8 hours** or every nursing shift until discharge: temperature, blood pressure, heart rate, respiratory rate, O2 saturation (including oxygenation method). Once the patient has been discharged, a single measurement of vital signs will be made at the study visits detailed in the table.<br><br>4 The electrocardiogram will be performed during the baseline visit and on days 1, 2, 3, 4, 5, 6, 7, 15 and 31 from the start of treatment with plitidepsin. The PR interval and the QT interval will be measured.<br>5 Complete blood count on days 1, 2, 3, 4, 5, 6, 7, 15 and 31.<br><br>6 Basic biochemistry on days 1, 2, 3, 4, 5, 6, 7, 15 and 31. It will include transaminases, bilirubin, alkaline phosphatase, LDH, CK, ferritin and troponin.<br><br>7 Coagulation on days 1, 2, 3, 4, 5, 6, 7, 15 and 31, including D-dimer.<br><br>8 C-reactive protein on days 1, 2, 3, 4, 5, 6, 7, 15 and 31.<br><br>9 The first time the patient tested positive for COVID-19 will be included as well. The test will be repeated in the 48 hours prior to administration of plitidepsin and on days 4, 7, 15 and 31, if negative detection does not occur before then. Qualitative PCR shall be obtained on nasopharyngeal exudate (preferably) or a sample from the lower respiratory tract.<br><br>10 A chest X-ray will be performed at baseline and subsequently based on clinical discretion.<br><br>11 Only fertile women.<br><br>12 Patients may receive other pharmacological treatments for COVID-19 at the discretion of the investigator 24 hours after administration of the last dose of plitidepsin.<br><br>13 Adverse events (serious and non-serious) will be recorded from signing of the informed consent up to 28 days after administration of the last dose of plitidepsin.<br><br>14 A safety visit will be made 28 days (+3 days) after the last dose of plitidepsin.<br>15 The viral load should be determined in a sample obtained from nasopharyngeal exudate (preferably) or a sample from the lower respiratory tract.

11. ADVERSE EVENTS

Safety information in clinical studies must be collected efficiently and consistently. Adverse events should be identified and reported promptly, so that potential risks to patients can be determined and regulatory requirements for reporting of adverse events can be met.

11.1. Definitions

11.1.1 Adverse Event (AE)

An AE is any undesirable event that occurs to a patient undergoing clinical research, temporarily associated with the use of a drug, whether or not it is deemed to be related to the investigational products. Therefore, an adverse event can be any unintended and unfavourable sign (including an abnormal analytical result), symptom or disease that is temporarily associated with the use of a drug, whether or not it is deemed to be related to it. Pre-existing conditions that worsen during the study will be reported as Adverse Events.

AEs include pre or post-treatment events that occur as a consequence of study procedures (e.g. invasive procedures or modification of the patient's previous medication).

11.1.2 Serious Adverse Event (SAE)

An SAE is an undesirable medical event that, at any dosage:

- Causes death.
- Is life-threatening (NOTE: The term “life-threatening” refers to the investigator's judgement that the patient is at risk of death as a consequence of said event. However, this term does not refer to the fact that if the AE had been more intense, it would have placed the patient at imminent risk of death).
- Causes hospitalisation or prolongs a previous hospitalisation (NOTE: Hospitalisation is generally understood to mean that the patient has stayed in the hospital or emergency department for at least 24 hours. Complications that occur during hospitalisation are AEs. If a complication prolongs hospitalisation or meets any of the other severity criteria, then the event will be considered an SAE. When in doubt as to whether or not “hospitalisation” has occurred, the AE will be considered serious. Hospitalisation to carry out a scheduled treatment of a disease prior to the subject entering the study and that has not worsened with respect to their baseline situation is not considered an SAE). Hospitalisation due to social reasons is not considered an AE.
- Causes impairment or disability (NOTE: Impairment involves significant impact on a person's ability to carry out their daily tasks, but not that caused by minor medical conditions such as headaches, nausea, vomiting, diarrhoea, influenza or

accidental trauma (such as a ankle sprain), which can hinder or interfere with activities of daily living, but do not significantly impair them).

- Results in a congenital anomaly or birth defect.
- Is medically important (that is, a medically important adverse event may not be immediately life-threatening or result in immediate hospitalisation or death. However, if it is determined that the event may harm the patient and/or may require intervention to prevent one of the outcomes specified above, this event should be reported as serious).

11.1.3 Adverse reaction

Any harmful and unintended response to a medication, including adverse reactions resulting from any use outside the terms of the marketing authorisation, abuse and error in the medication. There is a confirmed causal relationship with it.

11.2. Characteristics of an adverse event

11.2.1. Severity

See definition of Serious Adverse Event (11.2 Serious Adverse Event (SAE)).

11.2.2 Determination of causality

The investigator is required to establish the causal link between the investigational drug and the adverse event (whether serious or not). A “reasonable possibility” is proposed to define cases where there are facts/evidence or arguments to suggest a causal link. The investigator will use clinical judgement to determine the relationship.

Alternative causes, such as the natural history of underlying diseases, concomitant treatment, other risk factors and the temporal link between the event and the investigational drug will be considered and investigated. The investigator will also consult the investigator's brochure for this evaluation.

There may be situations when an AE occurs where the investigator has minimal information to include in the initial report for the Sponsor. However, it is very important that the investigator always make a causality assessment for each event before submitting the data to the Sponsor. The investigator may change their opinion on causality based on information that arises during follow-up, so the report on the AE must be modified. Assessment of causality is one of the criteria that determine whether or not a case meets the reporting criteria for referral to the health authorities.

11.2.3 Expectability

An unexpected adverse reaction is any that generates an unexpected adverse response that is related to the administration of the study drug at any dose and that is not consistent with the information in the investigator's brochure for the investigational product.

The sponsor will classify Adverse Reactions as expected or unexpected according to the Reference Safety Information included in the investigator's brochure.

11.2.4 Severity

The intensity of AEs will be evaluated using the CTCAE scale v. 5.0 (see Annex 6). If a CTCAE code does not exist, the following guide will be used:

Grade	Description
0	No adverse event
1	Mild; asymptomatic or mild symptoms; only requires clinical or symptomatic observation
2	Moderate; minimal, local or non-invasive intervention
3	Severe or medically important, but not immediately life-threatening; hospitalisation or extension of hospital stay
4	Life-threatening; requires urgent intervention
5	Death related to the adverse event

The grading of AEs is based on specific clinical criteria and requires evaluation by the site’s investigator.

11.2.5 Additional considerations

Alterations in laboratory parameters (haematology, biochemistry or urine analysis) should be recorded as an AE or SAE, as well as abnormal results of other tests (such as ECGs, radiological tests, measurements of vital signs), including those that worsen from baseline values, which according to the investigator's medical and scientific criteria are considered clinically relevant.

In contrast, clinically significant changes in safety parameters associated with the study disease will not be classified as an AE or SAE unless the investigator determines that they are more serious than expected taking into account the patient's condition.

11.3. Reporting procedures

11.3.1. Reporting of Adverse Events (AEs)

During the visits, the investigator will try to obtain, by means of examination or questioning the patient directly, all the information related to adverse events that have occurred.

All information regarding adverse events should be recorded in the corresponding section of the case report form. All adverse events occurring during the period covered will be recorded, from the moment the patient is included in the study (from signing of the consent form) up to 28 days after the last dose of the investigational product. When one or more signs or symptoms correspond to a disease, the main diagnosis or

syndrome will be reported. All adverse events will be followed up until resolution, stabilisation or until it is determined that study treatment or participation in the study was not the cause of the event. All adverse events that continue at the end of the study period should also be followed up to determine their final outcome.

11.3.2. Reporting Serious Adverse Events (SAEs)

All serious adverse events that occur during the trial, whether or not they are related to the study medication, must be reported immediately by email or by fax (completing the SAE form) within 24 hours of being reported by the investigator:

ATT. SAFETY DEPARTMENT
Fax[redacted]
Email[redacted]

The investigator will record the information regarding the SAE on the corresponding form. At least these **4 elements are mandatory** in order to report any adverse event:

- Name or identifier of the person **reporting** the SAE
- **Patient** number or identifier
- The **suspected drug** used in the trial: dose, route of administration, start and end date of treatment
- Information related to the **Adverse Event**:
    - Adverse Event term
    - Severity criteria
    - Assessment of causality

11.3.3. Reporting of Serious Adverse Events to the Health Authorities

It is the responsibility of the sponsor to notify the Health Authorities of all suspected serious and unexpected adverse reactions that have occurred in the clinical trial. This will be communicated within the time limits established in accordance with current regulations.

11.3.4. Monitoring of AEs and SAEs

After the initial AE or SAE report, the investigator is required to follow up on each case and obtain more information about its status. All AEs and SAEs documented on previous visits should be reviewed on subsequent visits. All AEs and SAEs must be evaluated until their resolution. This applies to all patients, including those who withdraw early.

The investigator will include in the follow-up any additional investigation that may clarify the nature and/or cause of the AE or SAE. This may include additional laboratory tests or investigations, histopathological examinations or consultation with other healthcare

professionals. Any co-investigator may request that the investigator carry out additional evaluations to clarify the nature and/or cause of the AE or SAE. If the patient dies during participation in the study or during an established follow-up period, the investigator will provide a copy of any post-mortem findings, including histopathology, that are requested.

11.3.5. AEs or SAEs that occurred after the end of the study

Post-study AEs or SAEs are defined as any event occurring outside the follow-up period defined in the protocol. The investigator is not required to actively search for the onset of AEs or SAEs that have occurred in patients who have participated in the clinical trial in the past. However, if at any time after a patient withdrew from the study the investigator should become aware of the existence of any AE or SAE, including the patient's death, that is deemed to be related to the study drug, the investigator should promptly notify the sponsor.

11.3.6. Pregnancy and breastfeeding

Study patients should be advised to notify the investigator immediately if they become pregnant during the study. Pregnant patients will stop receiving the study treatment. Patients will also be advised to report any pregnancy that occurred after the last visit if they believe conception occurred during their participation in the study.

A pregnancy in itself is not an AE unless there is a possibility that the investigational drug has interfered with the effectiveness of any of the contraceptive methods used. However, the investigator must notify pregnancies in accordance with the procedures and deadlines described for SAE reporting (section 11.3.2). The pregnancy reporting form should be used.

The pregnancy reporting forms must be submitted within 24 hours after the investigator becomes aware of the event:

ATT. SAFETY DEPARTMENT
Fax: [redacted]
Email: [redacted]

In accordance with local data privacy laws, the sponsor may request the contact information of the subject's doctor in order to follow the pregnancy until the outcome of the birth.

The pregnant patient or the patient’s pregnant partner will be monitored until the end of the pregnancy. Any complications during pregnancy or lactation should preferably be reported as AEs. The outcome of the pregnancy must be reported on the pregnancy reporting form. Any miscarriage, stillbirth, birth defect/abnormality, death or other serious conditions of the baby should be reported and followed up as if it were an SAE.

11.3.7. Overdose

An overdose is defined as any dose greater than the highest dose included in the protocol. Consult the infusion guide for detailed instructions on preparing, storing and administering the investigational drug.

Any overdose should be noted in the CRF as an Adverse Event and the investigator must report them in accordance with the procedures and deadlines described for SAE reporting (section 11.3.2) if the overdose is related to a serious adverse event.

12. STATISTICAL CONSIDERATIONS

12.1. General considerations

This section describes the main analyses that will be carried out. The statistical analysis plan to be approved by the sponsor will include additional information on analyses, management of unavailable data and analytical methods. All data will be analysed with the SAS statistical analysis system. All analyses will be carried out mainly by descriptive statistical methods. Continuous endpoints will be described using mean and standard deviation, median, minimum, maximum, Q1 and Q3. Categorical endpoints will be described with frequencies and percentages as well as the exact 95% confidence interval of the relevant study variables. All results will be presented separately by treatment arm.

The final statistical analysis that will be the basis of the final report of the study will be done on the total population of 45 patients once the extension of the study has been completed. The data of the first 27 patients will be analysed in an intermediate statistical analysis.

12.2. Sample calculation

The primary objective of the study is to evaluate the safety and toxicology profile of plitidepsin at each dose level administered once a day for 3 consecutive days.

Groups of 9 patients will be included and will be randomly assigned to each of the 3 arms. Depending on the safety analysis of each group of 3 patients included in each arm, it will be decided whether it is possible to include 3 more patients in the same arm, up to a maximum of 9 patients in each arm and a maximum of 27 patients in the study.

During the extension period of the study that will start after the recruitment of the 27 patients initially planned, an additional 18 patients will be included (6 at each dose level).

12.3. Analysis populations

Safety population:

The population for the safety analysis will consist of all randomised patients who receive at least one dose (complete or not) of plitidepsin.

Efficacy population:

Patients are considered evaluable for efficacy if they have received all three doses of treatment and have completed a follow-up period of at least 2 weeks. Patients who do not complete the treatment period because the disease has evolved unfavourably will also be evaluable for efficacy.

12.4. Analysis of the primary endpoint

The safety analysis will be performed in the safety population, that is, in patients who have received at least one dose of treatment. Patients will be analysed in the administered treatment arm.

Safety and tolerability analyses will be presented by treatment arm as well as by treatment arm according to age group (<65 years; ≥65 years). Adverse events will be coded using the latest version of the MedDRA dictionary and assessed using the NCI-CTCAE criteria v. 5.0.

The number and percentage of patients presenting the following ≥ grade 3 adverse events will be provided, according to the NCI-CTCAE criteria v. 5.0 at 3, 7, 15 and 31 days after the start of study treatment:

- Haematological
  - Neutropenia
  - Thrombopenia
  - Anaemia
  - Lymphopenia
- Non-haematological
  - CPK increase
  - ALT and/or AST increase
  - Total bilirubin or direct bilirubin increase
  - Neurotoxicity
  - QT-QTc interval prolongation
- Other ≥ grade 3 adverse events

- The number and percentage of patients in whom treatment cannot be completed and the reasons will be provided.

- The number and percentage of patients with AEs and SAEs will be provided at 3, 7, 15 and 31 days.

- The evolution of haematological and non-haematological parameters from the baseline visit on days 3, 7, 15 and 31 will be provided.

- The number and percentage of patients with ECG abnormalities on days 1, 2, 3, 4, 5, 6, 7, 15 and 31 will be provided. Any alterations detected will be described in detail.

12.5. Analysis of secondary endpoints

12.5.1. Efficacy assessments

Efficacy analyses will be performed on the efficacy population defined above. For the evaluation of efficacy endpoints, the following analyses will be carried out separately for each dosage level:

- The evolution of the viral load from the baseline visit on days 3, 4, 7, 15 and 31 will be provided.
- The time to negative detection of COVID-19 by PCR will be calculated as the time that elapses in days from the date of randomisation to the first date of PCR with a negative result. The time to negative detection will be analysed following the Kaplan-Meier methodology, providing the median and the corresponding 95% confidence interval.
- The number and percentage of patients who die 7, 15 and 31 days after starting the treatment with plitidepsin shall be described.
- The number and percentage of patients who require mechanical ventilation and/or ICU admission at 7, 15 and 31 days will be provided.
- The number and proportion of patients requiring invasive mechanical ventilation shall be stated.
- The number and proportion of patients requiring non-invasive mechanical ventilation shall be stated.
- The number and proportion of patients requiring oxygen therapy shall be stated.

12.5.2. Secondary safety endpoints

The number and percentage of patients who present an adverse event during the study will be provided. The lists per patient of all adverse events that occurred during the study will be presented, indicating the severity, relationship and intensity.

Adverse events per patient (number and percentage) will be provided, calculating the maximum grade for each of the events recorded throughout the study for each of the patients. The adverse events analysed will be the ones described in the corresponding section of the case report form.

These analyses will be performed in the same way for new adverse events that the investigator deems related to the study drug. If the relationship of an adverse event with the study medication cannot be ruled out, it will be considered to be related.

The number and percentage of patients who present a serious adverse event during the study will be provided. The lists per patient of all serious adverse events that

occurred during the study will be presented, indicating the severity, relationship and intensity.

12.5.3. Interim safety analysis

Given the characteristics of the study, it is planned to carry out continuous safety analyses up to 12 days after the last administration of plitidepsin for each patient evaluable for safety in each arm of each group of 9 patients. These analyses will be carried out for the 3 patients included in each arm of each cohort before continuing the inclusion of patients in the study.

This analysis will be purely descriptive and the data will be presented by treatment arm.

The analyses to be provided will be identical to those detailed in section 12.4 for the main objective.

The sponsor will assess the results of the intermediate analyses with the Spanish Agency of Medicines and Medical Devices.

13. DATA MANAGEMENT AND QUALITY ASSURANCE

13.1. Clinical trial monitoring and audits

The sites participating in the study will be supervised by monitors appointed by the sponsor, with specific training for this clinical trial, making periodic visits to the site before, during and at the end of the trial. These follow-up visits will be carried out in accordance with the ICH E6 (R2) guidelines. Visits can be supplemented by telephone contact or written communication.

Priority will be given to centralised monitoring and/or remote monitoring of the participating sites, avoiding overloading the site staff with tasks or reviewing source data and postponing, as far as possible, the verification of source data until it is possible to access the medical history directly. The sponsor will agree the conditions for such monitoring with the participating sites and teams.

Procedures will be established to ensure the quality of any aspect related to the trial.

13.2. Audits

The sponsor may carry out periodic audits of ongoing clinical studies. Audits are independent of, and separate from, routine monitoring or quality control functions and are conducted to ensure compliance with the protocol, applicable regulations and ICH E6 (R2) guidelines.

During the clinical trial, the Quality Department of Pharma Mar SA or external auditors contracted by the sponsor may make audit visits to the site (ICH Topic E6 Guideline for GCP, Section 1.6).

13.3. Inspections

Participation in this clinical trial implies acceptance of possible inspection by national or foreign regulatory authorities.

13.4. Reporting of serious breaches

Serious breaches of the authorised protocol or of Royal Decree 1090/2015 that occur in Spain must be reported by the sponsor without undue delay and no later than seven calendar days from the detection of the breach to the Spanish Agency of Medicines and Medical Devices (AEMPS) and the CEIm.

For this purpose, a serious breach will be defined as any non-compliance that may significantly affect the safety and rights of the study subjects or the reliability and integrity of the data generated in the clinical trial.

Only serious breaches will be reported to the AEMPS and the CEIm, and breaches that do not constitute a serious breach will not have to be reported.

Each investigator has to document and explain in the clinical history of the subjects any breach to the approved protocol and/or Royal Decree 1090/2015. Investigators can implement a protocol breach to eliminate an immediate safety risk to the subject without prior CEIm approval, but such a breach must be reported to the study monitor within one business day. These incidents will be evaluated and, if appropriate, an amendment to the protocol will be prepared.

The monitor will document breaches during monitoring visits. The monitor will notify the investigator of the breach during the visit and the “Breach Form” document will be completed and signed by the investigator and monitor.

13.5. Data management

APICES will be responsible for processing and quality control of the data. Data management will be performed according to the Data Management Plan, standard operating procedures and the current regulations.

Data management based on Good Clinical Practice (GCP) standards refers to the activities defined to achieve safe routines for the efficient input of information in the study database, avoiding errors. Routines include procedures for CRF handling, application design and management, data entry and verification, data validation, database quality control and documentation of activities performed, including information on discrepancies found during the process. The database, data input screens and programming will be designed by APICES in accordance with the clinical trial protocol.

13.6. Electronic Case Report Forms (eCRF)

The collection of study data will consist of the electronic recording of all the information required by the eCRF. APICES will provide the eCRF.

All investigators agree to keep eCRFs filled in with truthful and accurate information as well as source documents that are part of medical records.

All the requested information must be completed in each patient's eCRF. If a patient is included in the study but does not receive treatment (e.g. screening error), only minimal data such as demographics and date of informed consent will be collected in the eCRF, as well as the reason for the screening error. In general, no queries will be issued for the data of these patients indicated in the eCRF as “Not done” or “Not available”.

For the patients included in the study, the information from the source documents will be included in each patient's eCRF by the staff of the research team of the site for this study and this information will be supervised by an APICES monitor. If the information requested on any variables is not available or does not apply, the reason must be included. Data should not be missing unless otherwise stated. Modifications to the eCRF must be made following the procedure described in the eCRF user guide and will be recorded in the eCRF.

Each completed eCRF must be reviewed, signed and dated by the Principal Investigator of the study without delay. The completed eCRF will be reviewed by the study monitor as soon as possible after completion. The site will be provided with an electronic copy of the final, approved and signed eCRF, which must be stored in the corresponding file.

13.7. Web-based electronic CRF

Clinical data (including adverse events and concomitant medication) will be entered into an application designed to collect study data. Access to the eCRF is password protected and includes internal quality controls, such as automatic data entry range controls, to identify inconsistent, incomplete or inaccurate data. The clinical data will be entered in the application based on the source documents. The profiles and data entry permits for the eCRF at each participating site will be established in advance. Only study staff from each site designated by the principal investigator will be authorised to enter the data in the eCRF. The principal investigator and the research team will receive the necessary training to complete the eCRF and will be informed, before the start of the study and before any study data are entered in the eCRF, of the security measures that they must take into account.

13.8. Data recording in the electronic CRF

All data must be entered in Spanish. The eCRFs should always reflect the latest observations of the patients participating in the study. Therefore, eCRFs should be completed as soon as possible during or after the patient's visit. The principal

investigator of each participating site must verify that all the data entered in the eCRFs are accurate and correct.

If some evaluations are not carried out, or if certain information is not available, not applicable or unknown, the investigator or any other authorised person must state it in the eCRF. The investigator will approve the data by means of an electronic signature, and this approval will serve to confirm the accuracy of the recorded data.

13.9. Query management

The monitor will review the eCRFs and evaluate their completion and consistency. Each eCRF will be compared against the respective source documents to ensure that there are no discrepancies in critical data. The investigator or other authorised personnel will carry out the data entry, corrections and modifications. The monitor cannot fill in data in eCRFs.

Once the clinical data of the eCRF have been recorded in the application, the corrections made to each of the variables will be recorded in the audit trail (e.g. the reason for any change, the name of the person who made the change, the time and date). If additional corrections to the data are required, the monitor in charge at the site or the data manager will include a query in the eCRF. The authorised site personnel will answer the queries generated in the application. This entire process will be recorded in the audit trail along with the name of the person who made the change, the time and the date.

13.10. Source documents

The eCRF is essentially considered a data entry form and should not replace the original medical records (or source document) unless otherwise specified in the study protocol. Source documents are all documents used by the investigator or the hospital that relate to the patient's medical history, which verify the patient's existence, the inclusion and exclusion criteria, and all the records that justify the patient's participation in the study. These include patient enrolment and randomisation, medication accounting record, analyses, laboratory tests, reports, dispensed material records for distribution, patient file, etc.

Each investigator is responsible for maintaining source documents. These will be available for inspection by the study monitor at each monitoring visit. All additional documentation completed in the eCRF, such as laboratory data, must be clearly identified with the study, visit and patient number. Any personal information (e.g. subject's name, initials) must be removed or redacted to preserve patient confidentiality.

13.11. User identifier

In each record included in the eCRF, the user's identification will be automatically added through their unique ID. The data for each of the patients included in the study

will be electronically signed by the investigator to document their review and confirmation that the data are accurate. This will be done using the Investigator's ID and password; the date and time of signing will be automatically added at the time of the electronic signature. Should it be necessary to modify data in an eCRF, the correction must be made according to the procedures of the software used.

13.12. Change control

To comply with regulatory requirements, eCRF data will be electronically filed in the file of each participating site. All changes are recorded in a protected audit trail, and it will be necessary to enter the reason for the change. Once all data have been entered, verified and validated, the database will be locked to prevent further changes to the study data.

13.13. Documentation storage

Essential documents and eCRF data should be kept for 25 years after the end of the trial, or for a longer period if other requirements apply, such as if the study is presented as a basis for drug registration in which Annex I of Royal Decree 1345/2007, of October 11, must be complied with.

The clinical history of the test subject must be kept in accordance with the provisions of Law 41/2002, of November 14, and according to the maximum period allowed by the hospital, institution or private practice.

14. ADMINISTRATIVE CONSIDERATIONS

The following administrative aspects are intended to guide the investigators participating in the study, but may be subject to changes based on the sponsor's procedures, legislation, or working documents or guidelines.

14.1. Legal considerations

This clinical trial will be carried out in accordance with the ethical principles in the Declaration of Helsinki developed by the World Medical Association (WMA) and following the standards of good clinical practice and applicable legislation.

The staff involved in conducting this trial will be trained and have sufficient experience to perform the necessary tasks.

The study will be carried out in accordance with the protocol. The protocol, any amendment and the informed consent form will be approved by a CEIm before the start of the study, in accordance with current legislation.

The decision of the CEIm on the execution of the study will be stated in writing. The investigator and/or sponsor are responsible for keeping the CEIm informed of any

relevant information about the study drug. All protocol amendments will be agreed upon by the sponsor and the investigator.

Administrative changes to the protocol are considered minor corrections and/or clarifications that have no impact on the way the study will be conducted.

14.2. Review by the Drug Research Ethics Committee

The ICH guidelines require the approval of the health authorities and a favourable opinion from an accredited Clinical Research Ethics Committee (in Spain CEIm) before patients can be included in clinical trials. Before the start of the study, the protocol, the informed consent form, the materials that will be used for patient recruitment (if applicable) and any other written information regarding the trial that is provided to the patient will be approved by the CEIm. The clinical trial can only be started when both the Health Authorities and the CEIm have considered that the expected benefits for the study patients and society justify the risks; in addition, this criterion must be continuously reviewed to continue with the trial with guarantees.

APICES will obtain the favourable opinion of the CEIm on behalf of the sponsor and the investigators. All regulatory approvals will be signed by the President of the CEIm, or the person appointed by them, and must identify the name and address of the CEIm, the trial protocol using the protocol title and/or code, and the date of approval or date on which the favourable opinion is granted. Documentation related to authorisation by the CEIm and the health authorities and CEIm compliance with ICH E6 (R2) guidelines will be kept at the site and will be available for review.

14.3. Protocol modifications

Any change to the approved protocol will require an amendment to the protocol. The investigator should not make any changes to the study without the favourable opinion of the CEIm and the authorisation of the health authorities (if applicable), except when necessary to eliminate an imminent and obvious risk for the patients or in the case of administrative changes. Modifications to the protocol to eliminate an imminent and obvious risk can be implemented immediately, but must subsequently be documented in an amendment, notified to the CEIm and the relevant health authorities within the required period.

Any major modifications to the protocol must be sent to the CEIm and the health authorities for approval before the changes proposed in the amendment can be applied. Depending on the magnitude of the change, recruitment could be temporarily suspended.

The sponsor does not have to notify minor modifications to the health authorities or the CEIm. However, a record will be kept of minor modifications, filed with the study documentation and notified when sending another notification or when a major

modification is submitted. Documentation related to minor modifications will be available for inspection in the study file at the site or at the sponsor's facilities.

14.4. Informed consent

Before the patient is included in the study or before any specific procedure is carried out, informed consent must be obtained in writing from the patient or their legal representative, in accordance with ICH E6 guidelines. During the study, blood and serum samples will be collected before treatment and 7 days after treatment (two 6 ml tubes) to carry out a biomarker sub-study for which patients will have to give their consent.

The samples will initially be kept at each of the participating centres and will be sent for analysis to a central laboratory not yet selected by the sponsor, within the European Union.

For obtaining and documenting informed consent, the investigator must comply with applicable legislation, and must adhere to the standards of Good Clinical Practices as well as the ethical principles of the Declaration of Helsinki. The patient information leaflet and the informed consent form and any revisions thereof must be approved by the CEIm before being given to patients who are deemed eligible to participate in the study or to their legal representatives.

The sponsor will provide the research team of each participating site with the patient information leaflet and the informed consent form approved by the CEIm.

Before performing any study procedure on the patient, it is the responsibility of the principal investigator (or person appointed by them) to obtain consent, freely given in writing, from the patient or their legal representative after adequately explaining the objectives, methods, expected benefits and potential risks of the study and before any protocol-specific screening procedure is performed or study drug is administered. Patients or their legal representatives should have the opportunity to ask questions and receive the requested information, and they will have sufficient time to decide whether or not they want to participate in the study. Once the investigator makes sure that the patient understands the implications of participating in the study, they will be asked to sign the informed consent in order to obtain their consent to participate in the study.

The informed consent form must be personally signed and dated by the patient or their legal representative and by the study doctor who carried out the informed consent process (Principal Investigator or appointed person).

The acquisition of written informed consent must also be documented in the patient's medical history.

The investigator must provide a copy of the signed informed consent form to the patient or their legal representative. Another original copy of the document will be filed in the investigator's study file at the site.

If the informed consent form is reviewed and modified during the study, active participating subjects must sign the document reviewed and approved by the CEIm.

The patient participating in the clinical trial, or their legal representative, can withdraw consent at any time, without giving any explanation and without this entailing any harm to the patient.

14.5. Confidentiality

The collection and processing of personal data of the patients included in this clinical trial will be limited to the data that are necessary to investigate the efficacy, safety, quality and usefulness of the study medication used in this trial.

It is the investigator's responsibility to keep sufficient information to allow patient identification.

The trial monitor, sponsor auditors, CEIm and Competent Authorities should have direct access to all trial-related information and agree to keep the identity of study patients confidential.

The data must be collected and processed with the appropriate precautions to guarantee confidentiality and compliance with the current legislation regarding data privacy.

Explicit consent of the patients or their representatives for the treatment of personal data will be obtained before data collection, if necessary, and this consent will include the transfer of the data to other companies and countries.

Pharma Mar SA will comply with Regulation (EU) 2016/679 of the European Parliament and of the Council of 27 April 2016 on the protection of natural persons with regard to the processing of personal data and on the free movement of such data.

14.6. Insurance policy

The sponsor has contracted an insurance policy to cover the responsibilities of the investigators and the other parties participating in the study in accordance with the applicable regulatory requirements.

14.7. Publications

Before the investigators of this study send a manuscript or abstract for publication or publicly disclose information related to the study drug or products, they will provide this publication to Pharma Mar SA at least 60 days beforehand for review and approval, to guarantee that confidential data and data ownership are protected.

If Pharma Mar SA determines that any patentable aspect is disclosed in the proposed publication or communication, it will withhold the publication or communication for a period of time that it deems appropriate. If the study is part of a multi-centre study, the

first publication of the study will be carried out with the global results of the study, taking into account the investigators and institutions of all participating sites that have contributed data, analysis and comments. However, if such publication of the global results is not presented within 12 months after the end of the study in all the sites, the study could be published individually according to the procedure established above.

The order of the co-authors will reflect the relative contribution of each of them in the development of the study and in the analysis. In general, the first author will be the investigator who has recruited the largest number of patients with information that is ultimately available for data analysis. Pharma Mar SA staff who have fully participated in the study should be considered for co-authorship in the publication.

15. ACCEPTANCE OF THE PROTOCOL BY THE SPONSOR

Clinical trial title: Multicenter, randomized, parallel and proof of concept study to evaluate the safety profile of three doses of Plitidepsin in patients with COVID-19 requiring hospitalization.

Protocol code: APLICOV-PC (APL-D-002-20)

Version (number and date): Version 7.0 of 29 September 2020

Position (pre-printed name)	Signature	Signature Date (DD-Mmm-YYYY)
Position (pre-printed name)	Signature	Signature Date (DD-Mmm-YYYY)
Position (pre-printed name)	Signature	Signature Date (DD-Mmm-YYYY)

16. ACCEPTANCE OF THE PROTOCOL BY THE PRINCIPAL INVESTIGATOR AND COLLABORATORS

Clinical trial title: Multicenter, randomized, parallel and proof of concept study to evaluate the safety profile of three doses of Plitidepsin in patients with COVID-19 requiring hospitalization

Protocol code: APLICOV-PC (APL-D-002-20)

Version (number and date): Version 7.0 of 29 September 2020

I have read and carefully reviewed the study protocol. Having read and understood the requirements and conditions of the study protocol, I agree to carry out the clinical study according to the international principles of Good Clinical Practice and the requirements of the regulatory authorities for the verification of the original documents and the audit/inspection of the study. I agree to use the study material, including the medication, only as specified in the protocol.

I understand that the changes made to the protocol must be made in the form of an amendment with the sponsor's prior written approval. I understand that any breach of the protocol may lead to early termination of the study.

Signature of the Principal Investigator

Signature Date
(DD-Mmm-YYYY)

Investigator's name (uppercase)

Signature of the Co-investigator

Signature Date
(DD-Mmm-YYYY)

Investigator's name (uppercase)

Signature of the Co-investigator

Signature Date
(DD-Mmm-YYYY)

Investigator's name (uppercase)

17. BIBLIOGRAPHIC REFERENCES

1. Rinehart KL Jr, Gloer JB, Hughes RG et al. Didemnins: antiviral and antitumor depsipeptides from a Caribbean tunicate. Science. 1981 May 22; 212 (4497): 933-5.

2. Canonico PG, Pannier WL, Huggins JW, Rienehart KL. Inhibition of RNA viruses in vitro and in Rift Valley fever-infected mice by didemnins A and B. Antimicrob Agents Chemother. 1982 Oct; 22 (4): 696-7.

3. Losada, A., et al., Translation Elongation Factor eEF1A2 is a Novel Anticancer Target for the Marine Natural Product Plitidepsin. Sci Rep, 2016. 6: p. 35100.

4. Mateyak, M.K. and T.G. Kinzy, eEF1A: thinking outside the ribosome. J Biol Chem, 2010. 285(28): p. 21209-13.

5. Zhou, B., et al., The nucleocapsid protein of severe acute respiratory syndrome coronavirus inhibits cell cytokinesis and proliferation by interacting with translation elongation factor 1alpha. J Virol, 2008. 82(14): p. 6962-71.

6. Ma-Lauer, Y., et al., Influences of cyclosporin A and non-immunosuppressive derivatives on cellular cyclophilins and viral nucleocapsid protein during human coronavirus 229E replication. Antiviral Res, 2020. 173: p. 104620.

7. Cervantes-Barragan, L., et al., Dendritic cell-specific antigen delivery by coronavirus vaccine vectors induces long-lasting protective antiviral and antitumor immunity. mBio, 2010. 1(4).

8. Wang M, Cao R, Zhang L, et al. Remdesivir and chloroquine effectively inhibit the recently emerged novel coronavirus (2019-nCoV) in vitro. Cell Research 2020; 30: 269–271.

9. PharmaMar, data on file; VPT1992/2014.

10. PharmaMar, data on file; VPT2678/2014.

11. Maroun, J.A., et al., Phase I study of Aplidine in a dailyx5 one-hour infusion every 3 weeks in patients with solid tumors refractory to standard therapy. A National Cancer Institute of Canada Clinical Trials Group study: NCIC CTG IND 115. Ann Oncol, 2006. 17(9): p. 1371-8.

12. Nalda-Molina R, et al. Population pharmacokinetics meta-analysis of plitidepsin (Aplidin) in cancer subjects. Cancer Chemother Pharmacol. 2009 Jun;64(1):97-108. doi: 10.1007/s00280-008-0841-4.

Annex 4. Preparation guide for plitidepsin infusion

Preparation for PLITIDEPSIN intravenous infusion

Appropriate aseptic techniques must be used. **Aplidin 2 mg** must be reconstituted and further diluted prior to infusion.

Each vial of Aplidin 2 mg is reconstituted with 4 ml of the solution for reconstitution macrogolglycerol ricinoleate (polyoxyl 35 castor oil)/Absolute Ethanol/Water for Injection, 15%/15%/70% (v/v/v).

Instructions for reconstitution

A syringe should be used to inject the 4 ml of solution for reconstitution into the vial. Shake the vial until complete dissolution. The reconstituted solution results in a clear, colourless or slightly off-yellow solution and free of visible particles.

This reconstituted solution contains 0.5 mg/ml of plitidepsin. It requires further dilution and is for single-use only.

Instructions for dilution

The reconstituted solution should be diluted with sodium chloride 9 mg/ml (0.9%) solution for infusion or glucose 50 mg/ml (5%) solution for infusion for administration as an intravenous infusion.

For a dose of 1.5 mg plitidepsin: 3 ml of plitidepsin reconstituted solution should be withdrawn from the vial and added to a suitable infusion container containing 300 ml of sodium chloride 9 mg/ml (0.9%) or glucose 50 mg/ml (5%) solution for infusion.

For a dose of 2 mg plitidepsin: 4 ml of plitidepsin reconstituted solution should be withdrawn from the vial and added to a suitable infusion container containing 400 ml of sodium chloride 9 mg/ml (0.9%) or glucose 50 mg/ml (5%) solution for infusion.

For a dose of 2.5 mg plitidepsin: 5 ml of plitidepsin reconstituted solution should be withdrawn from the vial and added to a suitable infusion container containing 500 ml of sodium chloride 9 mg/ml (0.9%) or glucose 50 mg/ml (5%) solution for infusion.

Instructions for IV administration

Plitidepsin infusion solution should be administered by intravenous infusion via peripheral or central line using infusion sets with a 0.2 micron polyethersulfone (PES) or Nylon in-line filter to remove air bubbles caused by the surfactant and to minimise the risk of exposure to adventitious pathogens that may be introduced during solution preparation.

Plitidepsin infusion solution should be administered within a maximum of 6 hours after

preparation at room temperature and ambient indoor light. This 6 hours period should be inclusive of the product administration period.

If the infusion solution is expected to be administered later than 6 h post dilution, the infusion solution must be protected from light.

Infusion solutions for plitidepsin doses of 1.5, 2.0 and 2.5 mg should be IV infused through a pump device over one hour and half (1h, 30 min).

Please refer to the clinical protocol for specific details regarding the appropriate dosage and infusion time to be used.

Infusion systems COMPATIBILITIES

Plitidepsin infusion solution should be administered with an infusion set and a sterile, non-pyrogenic, low-protein-binding filter (with a pore size of 0.2 μm) using an automated infusion pump.

Plitidepsin infusion solution is compatible with:

- Glass and polyolefin containers (polyethylene, polypropylene and mixtures).
- PVC DEHP-free and polyolefin infusion sets (polyethylene, polypropylene and mixtures).
- Polyethersulfone and nylon in-line filters with pore sizes of 0.2 μm.
- Implantable venous access systems with titanium and plastic resin ports and with polyurethane or silicone intravenous catheters. Polyurethane intravenous catheters can be used as no adsorption has been observed under these circumstances.

Incompatibilities

Medical devices containing PVC plasticised with DEHP as the main component in contact with plitidepsin are not compatible due to leaching of DEHP.

Polyurethane infusion lines are not compatible due to high adsorption values of plitidepsin to the plastic material.

Annex 5. CYP3A4 inducers and inhibitors

INHIBITORS							
**1ª2**	**2B6**	**2C8**	**2C9**	**2C19**	**2D6**	**2E1**	**3ª4,5,7**
fluvoxamine<br>ciprofloxacin<br>cimetidine<br>amiodarone<br>fluoroquinolones<br>furafylline<br>interferon<br>methoxsalen<br>mibefradil	thiotepa<br>ticlopidine	gemfibrozil<br>trimethoprim<br>glitazones<br>montelukast<br>quercetin	fluconazole<br>amiodarone<br>fenofibrate<br>fluvastatin<br>fluvoxamine<br>isoniazid<br>lovastatin<br>phenylbutazone<br>probenecid<br>sertraline<br>sulfamethoxazole<br>sulfaphenazole<br>teniposide<br>voriconazole<br>zafirlukast	PPIs:<br>lansoprazole<br>omeprazole<br>pantoprazole<br>rabeprazole<br>chloramphenicol<br>cimetidine<br>felbamate<br>fluoxetine<br>fluvoxamine<br>indomethacin<br>ketoconazole<br>modafinil<br>oxcarbazepine<br>probenecid<br>ticlopidine<br>topiramate	bupropion<br>fluoxetine<br>paroxetine<br>quinidine<br>duloxetine<br>terbinafine<br>amiodarone<br>cimetidine<br>sertraline<br>celecoxib<br>chlorpheniramine<br>chlorpromazine<br>citalopram<br>clemastine<br>clomipramine<br>cocaine<br>diphenhydramine<br>doxepin<br>doxorubicin<br>escitalopram<br>halofantrine<br>histamine H1<br>receptor antagonists<br>hydroxyzine<br>levomepromazine<br>methadone<br>metoclopramide<br>mibefradil<br>midodrine<br>moclobemide<br>perphenazine<br>ranitidine<br>red-haloperidol<br>ritonavir<br>ticlopidine<br>tripelennamine	Diethyl-<br>dithiocarbamate<br>disulfiram	HIV Antivirals:<br>indinavir<br>nelfinavir<br>ritonavir<br>clarithromycin<br>itraconazole<br>ketoconazole<br>nefazodone<br>saquinavir<br>telithromycin<br>aprepitant<br>erythromycin<br>fluconazole<br>grapefruit juice<br>verapamil<br>diltiazem<br>cimetidine<br>amiodarone<br>NOT azithromycin<br>chloramphenicol<br>ciprofloxacin<br>delavirdine<br>diethyldithiocarbamate<br>fluvoxamine<br>gesture<br>imatinib<br>mibefradil<br>mifepristone<br>norfloxacin<br>norfluoxetine<br>star fruit<br>voriconazole

**INDUCERS**							
**1ª2**	**2B6**	**2C8**	**2C9**	**2C19**	**2D6**	**2E1**	**3ª4,5,7**
broccoli<br>Brussels sprouts<br>char-grilled meat<br>insulin<br>methylcholanthrene<br>modafinil<br>nafcillin<br>beta-Naphthoflavone<br>omeprazole<br>tobacco	phenobarbital<br>rifampin	rifampin	rifampin<br>secobarbital	carbamazepine<br>norethindrone<br>NOT pentobarbital<br>prednisone<br>rifampin	dexamethasone<br>rifampin	ethanol<br>isoniazid	HIV Antivirals:<br>efavirenz<br>nevirapine<br>barbiturates<br>carbamazepine<br>efavirenz<br>glucocorticoids<br>modafinil<br>nevirapine<br>oxcarbazepine<br>phenobarbital<br>phenytoin<br>pioglitazone<br>rifabutin<br>rifampin<br>St John's wort<br>troglitazone

The list of potent CYP3A4 inducers and inhibitors can be found at the following Internet address:

https://www.fda.gov/drugs/drug-interactions-labeling/drug-development-and-drug-interactions-table-substrates-inhibitors-and-inducers

Annex 6. NCI-CTCAE criteria

The common terminology criteria for the classification of adverse events version 5.0 (NCI-CTCAE v. 5.0) can be consulted at the following Internet address:

https://ctep.cancer.gov/protocolDevelopment/electronic_applications/docs/CTCAE_v5_Quick_Reference_8.5x11.pdf
