# Supplementary material for: Preclinical and randomized phase I studies of plitidepsin in adults hospitalized with COVID-19
Source: Life Sci Alliance. 2022 Jan 10;5(4):e202101200. doi: 10.26508/lsa.202101200 (PMC8761492; doi:10.26508/lsa.202101200)
Supplement: Supplementary file 5 [file LSA-2021-01200_Supplemental_Data_2.docx]

Supplementary Appendix 3: Pre-clinical and randomized phase I studies of plitidepsin in adults hospitalized with COVID-19

José F. Varona^*^, Pedro Landete, Jose A. Lopez-Martin, Vicente Estrada, Roger Paredes, Pablo Guisado-Vasco, Lucía Fernández de Orueta, Miguel Torralba, Jesús Fortún, Roberto Vates, José Barberán, Bonaventura Clotet, Julio Ancochea, Daniel Carnevali, Noemí Cabello, Lourdes Porras, Paloma Gijón, Alfonso Monereo, Daniel Abad, Sonia Zúñiga, Isabel Sola, Jordi Rodon, Júlia Vergara-Alert, Nuria Izquierdo-Useros, Salvador Fudio, María José Pontes, Beatriz de Rivas, Patricia Girón de Velasco, Antonio Nieto, Javier Gómez, Pablo Avilés, Rubin Lubomirov, Alvaro Belgrano, Belén Sopesén, Kris M. White, Romel Rosales, Soner Yildiz, Ann-Kathrin Reuschl, Lucy G. Thorne, Clare Jolly, Greg J. Towers, Lorena Zuliani-Alvarez, Mehdi Bouhaddou, Kirsten Obernier, Briana L. McGovern, M. Luis Rodriguez, Luis Enjuanes, Jose M. Fernández-Sousa, Nevan J. Krogan, José M. Jimeno^†^, Adolfo García-Sastre^†^

* Corresponding author. José F. Varona, MD, Departamento de Medicina Interna, Hospital Universitario HM Monteprincipe, HM Hospitales, Av. de Monteprincipe 25, Madrid, Spain, 28660. Email: [jfvarona@hmhospitales.com](mailto:jfvarona@hmhospitales.com). ORCID: 0000-0003-4764-9440.

^†^ These authors contributed equally to this work.

**Changes in the Conduct of the Study or Planned Analyses.**

**Changes to Protocol.**

A total of 7 protocol versions were submitted to Spanish Agency of Medicines and Medical Devices for evaluation.

The following substantial changes were identified in each protocol version:

- Protocol v2.0 dated 25 April 2020:
  - Deleted the following provision at the request of the Spanish Agency of Medicines and Medical Devices: “In the event that in any of the arms of the study, following the joint evaluation of the safety data of the three patients in each arm between the promoter and the Spanish Agency of Medicines and Medical Devices , it is advisable to discontinue the inclusion of patients, the inclusion of patients in the rest of the study arms until the objectives of the clinical trial are completed”
- Protocol v3.0 dated 13 May 2020: implemented the relevant amendment #1 (AR1)
  - Modified exclusion criterion for lymphopenia from <1200/µL to <1000/µL
  - Added provision to allow other pharmacologic treatments for COVID-19 to be administered starting 24 hours after administration of the last dose of plitidepsin
- Protocol v4.0 dated 18 June 2020: implemented the relevant amendment #6 (AR6)
  - Modified inclusion criterion to change timing from onset of symptoms to study inclusion from 6 days to 10 days
  - Modified exclusion criterion for D-dimer from >1500 ng/mL to >4 x ULN
  - Modified exclusion criterion for lymphopenia from <1000/µL to <800/µL
  - Deleted exclusion criterion for coagulation parameters outside normal limits, except D-dimer
- Protocol v5.0 dated 13 August 2020: implemented the relevant amendment #9 (AR9)
  - Prophylactic medication prior to plitidepsin infusion modified to add ondansetron 8 mg IV slow infusion and change from oral to IV dexamethasone 8 mg *[note that the dose of dexamethasone was calculated as 8 mg dexamethasone phosphate, which is equivalent to 6.6 mg dexamethasone base]*
- Protocol v7.0 dated 29 September 2020: additionally modified protocol v6.0 (dated 11 September 2020, which was not approved). The changes respect to protocol version 5.0 were described in the relevant amendment #12 (AR12). In brief:
  - Modified enrollment scheme so that once the first 3 patients at 2.5mg had been followed for 12 days without observed unacceptable toxicity, the last 9 patients would be enrolled, with 6 randomized 1:1 to the 2.0 mg and 2.5 mg cohorts and the remaining 3 included in the 2.5 mg cohort without randomization
  - Added an expansion cohort of 18 additional patients (6 per dose level) once the first 27 patients had been enrolled to allow collection of additional safety and efficacy data
  - Added an interim statistical analysis based on the first 27 patients enrolled and issuance of an interim study report
  - Added provision that if any of the arms of the expansion cohort 2 or more patients experience a grade ≥3 AE, the Sponsor will interrupt recruitment of additional patients at that dose level and higher and assess jointly with the Spanish Agency of Medicines and Medical Devices regarding continued recruitment.
  - Modified the antiemetic regimen as follows: “Ondansetron 4 mg oral will be administered every 12 hours until 48 hours after the last administration of plitidepsin”.
  - Added provision that the Sponsor will submit to the Spanish Agency of Medicines and Medical Devices weekly reports of all grade ≥3 AEs, regardless of relationship, for joint review

There were other substantial amendments to the study that did not affect the protocol:

- AR2, AR3, AR5, AR11: Expansion of Study Sites
- AR4: New Economic Memory
- AR7: New version of the Investigational Medicinal Product Drug IMPD v6.0 02/06/2020
- AR8: New Inform Consent Form (ICF) v5.0
- AR10: New Investigational Brochure (IB) v 2.0
- AR13: An additional informed consent form (ICF) is generated for sending samples to a central laboratory.

***Post-hoc* Analyses.**

- Categorization of COVID-19 Disease Severity at Baseline

The study protocol did not specify baseline assessments of COVID-19 disease severity using the definitions in the FDA Guidance for Industry *“COVID-19: Developing Drugs and Biological Products for Treatment or Prevention”* (<https://www.fda.gov/media/137926/download>), which are based on positive PCR test for COVID-19, clinical symptoms, and clinical signs (respiratory rate, heart rate, and saturation of oxygen (SpO_2_) at room air at sea level), i.e.,

- - Mild COVID-19: positive PCR test for COVID-19; and symptoms of mild illness that could include fever, cough, sort throat, malaise, headache, muscle pain, nausea, vomiting, diarrhea, and loss of taste or smell, without shortness of breath or dyspnea
  - Moderate COVID-19: positive PCR test for COVID-19; symptoms of moderate illness, which could include any symptom of mild illness or shortness of breath with exertion; and clinical signs suggestive of moderate illness, such as respiratory rate ≥ 20 breaths per minute, heart rate ≥ 90 beats per minute, with SpO_2_ >93% on room air at sea level;
  - Severe COVID-19: positive PCR test for COVID-19; symptoms suggestive of severe systemic illness, which could include any symptom of moderate illness or shortness of breath at rest, or respiratory distress; and clinical signs indicative of severe systemic illness, such as respiratory rate ≥30 breaths per minute, heart rate ≥ 125 beats per minutes, SpO_2_ ≤ 93% on room air at sea level or PaO_2_/FiO_2_ < 300;
  - Critical COVID-19: positive PCR test for COVID-19 and evidence of critical illness, defined as at least one of the following: (i) respiratory failure defined based on resource utilization requiring at least one of endotracheal intubation and mechanical ventilation, oxygen delivered by high-flow nasal cannula (heated, humidified oxygen delivered via reinforced nasal cannula at flow rates > 20 L/min with fraction of delivered oxygen ≥0.5), non-invasive positive pressure ventilation , ECMA, or clinical diagnosis of respiratory failure (i.e., clinical need for one of the preceding therapies but preceding therapies not able to be administered in setting of resource limitation); (ii) shock, defined by systolic blood pressure < 90 mm Hg, or diastolic blood pressure < 60 mm Hg or requiring vasopressors; or (iii) multi-organ dysfunction/failure.

However, assessments of disease severity were made *post-hoc* as follows. Per protocol, all patients had positive PCR tests for COVID-19 (performed at local laboratories) and non-invasive oximetry evaluations performed at baseline. For some patients, oximetry was not assessed at room air conditions, implying that these patients were receiving oxygen supplementation and, therefore, had at least moderate severity disease. To discriminate between moderate and severe disease categories, the PaO_2_/FiO_2_ ratio was estimated from SpO_2_.

The Ellis inversion of the Severinghaus equation was used to programmatically impute PaO_2_/FiO_2_ from non-invasive PaO_2_/SpO_2_ and to categorize APLICOV-PC patients as having moderate or severe disease, using the following rationale:

The SpO_2_/FiO_2_­ ratio has been proposed as a noninvasive surrogate for the PaO_2_/FiO_2_ ratio. (Khemani et al 2009, 2012; Lobete et al 1989; Pandharipande et al 2009; Rice et al 2007; Thomas et al 2010). To ensure equivalence, a noninvasive surrogate would require imputation of PaO_2_ from SpO_2_. The Ellis inversion (Ellis 1989) of the Severinghaus equation (Severinghaus 1979) was used to impute PaO_2_ from SpO_2_; this technique has been used previously in cohorts of non-intubated patients with pneumonia (Brown et al 2009; Dean et al 2012; Lanspa et al 2013). In recent work exploring different methods, the non-linearly imputed PaO_2_/FiO_2_ based on the Severinghaus equation outperformed linear and log-linear imputations. Additionally, the mortality associated with non-linearly imputed PaO_2_/FiO_2_ thresholds was closer to the mortality associated with measured PaO_2_/FiO_2_ thresholds (Brown et al 2016).

- Classification of Patient Status at Discharge Using a 6-Point Ordinal Scale.

Although not specified in the protocol (so not captured in the CRF), *post-hoc* analysis was performed to categorize the status of each patient at baseline, on Days 7, 15, 31, and at time of discharge using the following 6-point ordinal scale (Wang et al 2020):

1. Discharge (alive)
2. Hospital admission, not requiring supplemental oxygen
3. Hospital admission, requiring supplemental oxygen
4. Hospital admission, requiring high-flow nasal cannula or noninvasive mechanical ventilation
5. Hospital admission, requiring extracorporeal membrane oxygenation or invasive mechanical ventilation
6. Death

The number and percentage of patients with at least 1-point and at least 2-point improvements from baseline was calculated for Days 7, 15, 31 and date of discharge.

- Multivariate Analysis to Identify Factors Associated with Hospital Discharge on Day 15.

Two types of univariate logistic models were built, one with only the covariate and the other including both the covariate and intercept. When the intercept is not significant the valid associated p-values are univariate models containing only the single variable but when the intercept is significant the associated p-values are univariate models containing both parameters. As a second step, covariates with a p-value <0.10 and dose were included in a stepwise selection model. Variables included dose (1.5mg, 2.0mg, 2.5mg); age; time from symptom onset to first plitidepsin dose; number of comorbidities (0, 1, ≥2) and type of comorbidity, including cardiac disease, lung disease, asthma, kidney disease, diabetes, obesity, and hypertension; disease severity at randomization (mild, moderate, severe); baseline clinical signs including SpO_2_ no room air at baseline (<93% vs ≥93%), temperature, systolic blood pressure, diastolic blood pressure, heart rate, and respiratory rate; and baseline laboratory parameters of platelet count (x 10^9^/L), lymphocyte count (x 10^9^/L), ALT (xULN), AST (xULN), GGT (xULN), LDH (xULN), serum creatinine (µmol/L), D-dimer, ferritin, C-reactive protein, and log_10_ viral load.

- Multivariate Analysis to Identify Factors Associated with Hospital Discharge on Day 8.

A similar exploratory analysis was performed with this timepoint. Variables included in a logistic regression model were: dose (1.5mg, 2.0mg, 2.5mg); age; disease severity at randomization (mild, moderate, severe); and log_10_ viral load.

**References**

Brown SM, Jones BE, Jephson AR, Dean NC: Infectious Diseases Society of America/American Thoracic Society validation of the Infectious Diseases Society of America/American Thoracic Society 2007 guidelines for severe community-acquired pneumonia. Crit Care Med 2009; 37:3010-3016

Brown SM, Grissom CK, Moss M, et al: Nonlinear imputation of PaO2/FiO2 from SpO2/FiO2 among patients with acute respiratory distress syndrome. Chest 2016; 150:307-313

Dean NC, Jones JP, Aronsky D: Hospital admission decision for patients with community-acquired pneumonia: variability among physicians in an emergency department. Ann Emer Med 2012; 59:35-41

Ellis RK: Determination of PO2 from saturation. J Appl Physiol 1989; 67:902-902

Khemani RG, Patel NR, Bart RD III, Newth CJ: Comparison of the pulse oximetric saturation/fraction of inspired oxygen ratio and the PaO2 fraction of inspired oxygen in children. Chest 2009; 135:662-668

Khemani RG, Thomas NJ, Venkatachalam V: Comparison of SpO2 to PaO2 based markers of lung disease severity for children with acute lung injury. Crit Care Med 2012; 40:1309-1316

Lanspa MJ, Jones BE, Brown SM, Dean NC: Mortality, morbidity, and disease severity of patients with aspiration pneumonia. J Hosp Med 2013; 8:83-90

Lobete C, Medina A, Rey C, et al: Correlation of oxygen saturation as measured by pulse oximetry/fraction of inspired oxygen ratio with PaO2/fraction of inspired oxygen ratio is a heterogeneous sample of critically ill children. J Crit Care 2013; 28:538.e1-538.e7

Pandharipande PP, Shintani AK, Hagerman HE: Derivation and validation of SpO2/FiO2 ratio to impute for PaO2/FiO2 ratio in the respiratory component of the Sequential Organ Failure Assessment score. Crit Care Med 2009; 37:1317-1321

Rice T, Wheeler AP, Bernhard GR: Comparison of the SpO2/FiO2 ratio and the PaO2/FiO2 ratio in patients with acute lung injury or ARPD. Chest 2007; 132:410-417

Severinghaus JW: Simple accurate equation for human blood O2 dissociation computations. J Appl Physiol 1979; 46:599-602

Thomas NJ, Shaffer ML, Willson DF, Shih MC, Curley MA: Defining acute lung disease in children with the oxygen saturation index. Pediatr Crit Care Med 2010; 11:12-17

U.S. Department of Health and Human Services Food and Drug Administration Center for Drug Evaluation and Research (CDER) Center for Biologics Evaluation and Research (CBER). COVID-19: Developing Drugs and Biological Products for Treatment or Prevention Guidance for Industry. February 2021. <https://www.fda.gov/media/137926/download>

Wang Y, Zhang D, Du G, et al: Remdesivir in adults with severe COVID-19: a randomized, double-blind, placebo-controlled, multicentre trial. Lancet 2020; 395:1569-1578
